# Physical exercise restores adult neurogenesis deficits induced by simulated microgravity

**DOI:** 10.1038/s41526-024-00411-6

**Published:** 2024-06-21

**Authors:** Alexandra Gros, Fandilla Marie Furlan, Vanessa Rouglan, Alexandre Favereaux, Bruno Bontempi, Jean-Luc Morel

**Affiliations:** 1 CNRS, INCIA, UMR 5287, University Bordeaux, F-33000 Bordeaux, France; 2 CNRS, IMN, UMR 5293, University Bordeaux, F-33000 Bordeaux, France; 3 Centre National d’Etudes Spatiales, F-75001 Paris, France; 4 CNRS, IINS, UMR 5297, University Bordeaux, F-33000 Bordeaux, France; 5 Present Address: Department of Genetics & Evolution, 30 Quai Ernest-Ansermet, 1205 Geneva, Switzerland

**Keywords:** Neuroscience, Genetics

## Abstract

Cognitive impairments have been reported in astronauts during spaceflights and documented in ground-based models of simulated microgravity (SMG) in animals. However, the neuronal causes of these behavioral effects remain largely unknown. We explored whether adult neurogenesis, known to be a crucial plasticity mechanism supporting memory processes, is altered by SMG. Adult male Long-Evans rats were submitted to the hindlimb unloading model of SMG. We studied the proliferation, survival and maturation of newborn cells in the following neurogenic niches: the subventricular zone (SVZ)/olfactory bulb (OB) and the dentate gyrus (DG) of the hippocampus, at different delays following various periods of SMG. SMG exposure for 7 days, but not shorter periods of 6 or 24 h, resulted in a decrease of newborn cell proliferation restricted to the DG. SMG also induced a decrease in short-term (7 days), but not long-term (21 days), survival of newborn cells in the SVZ/OB and DG. Physical exercise, used as a countermeasure, was able to reverse the decrease in newborn cell survival observed in the SVZ and DG. In addition, depending on the duration of SMG periods, transcriptomic analysis revealed modifications in gene expression involved in neurogenesis. These findings highlight the sensitivity of adult neurogenesis to gravitational environmental factors during a transient period, suggesting that there is a period of adaptation of physiological systems to this new environment.

## Introduction

The increased duration and distance of future space missions may compromise the health of crew-members who will have to adapt to a highly challenging environment. Numerous environmental factors constitute stressors for astronauts: microgravity and altered gravity, exposure to ionizing particle radiation, social isolation, and other spaceflight hazards^1^. These stressful stimuli could affect physiological systems, triggering biological adaptive changes to re-establish the optimal homeostatic state. During spaceflights, astronauts report mental fatigue, disruptions of neurovestibular system, sleep and circadian rhythms, and difficulty concentrating and maintaining a high level of cognitive performance^2–4^. MRI investigations have revealed that the brain of astronauts undergoes a variety of changes as a consequence of spaceflight missions including changes in tissue volume and microstructure, cerebrospinal fluid distribution and dynamics, and functional connectivity^5–7^. Understanding how long-term exposure to reduced-gravity environments may influence the neuro-physiological processes and neuroplasticity, i.e., the ability of the brain to organize its structure and related functions in response to stressful environmental stimuli has crucial translational significance for the health and adaptation of astronauts.

Animal model research is essential to identifying and understanding the mechanisms underlying space-related neurobiological and behavioral changes that could affect mental health and performance of astronauts. Rodent models have been developed in laboratories for years, in particular, the hindlimb unloading (HU) model, which simulates microgravity on Earth^8^. This model reproduces in rodents the principal effects of microgravity observed in humans such as atrophy in unloaded muscles, bone mineralization loss^9^, and cardiovascular deconditioning^10^. Studies using HU models have shown that exposure to simulated microgravity (SMG) can cause modifications in neurotransmission^11–15^, monoamine distribution^16^, morphological and electrophysiological changes in hippocampal neurons^17–20^, as well as modifications in protein and gene expression in the hippocampus and cerebral cortex^13,15,19–24^, and suggested to underlie cognitive deficits^11,13,15,18–20^.

Learning and memory processes are supported by neuronal plasticity mechanisms, including adult neurogenesis^25^. In the mammalian brain, new neurons are continuously generated throughout life in restricted brain areas called neurogenic niches: the sub-granular zone of the dentate gyrus (DG) of the hippocampus where new dentate granule cells are generated, and the sub-ventricular zone (SVZ) where new neurons are generated to supply the olfactory bulb (OB) in interneurons^26,27^. The process of adult neurogenesis starts with neural progenitor cells or stem cells. These progenitor cells divide many times before specializing into different cell types, mostly neurons. Adult neurogenesis involves an initial period of neural proliferation, followed by a period of survival, selection and maturation over the course of several weeks^26,27^. This dynamic and finely-regulated process is highly dependent on the activity of neural networks and subject to modulation by various physiological and environmental stimuli^28^. The impact of spaceflight on adult neurogenesis has been little explored. It is well-known that irradiation impairs adult neurogenesis in the SVZ and DG^29–31^, making it a standard uninvasive method to delete new neurons in specific neurogenic regions of adult rodents^32^. Social isolation and/or exposure to an impoverished environment are also known to influence adult neurogenesis^33–35^. However, the impact of microgravity on adult neurogenesis remains understudied. In vitro, neural stem cells derived from the SVZ of HU mice present a reduced proliferation capability, an altered cell cycle and an incomplete differentiation/maturation^36^. In vivo, HU for 2 weeks reduces the number of proliferating cells in the SVZ^36,37^ and DG^37^. However, these studies were limited to proliferation and did not explore newborn cell survival and maturation.

Here, we explored the impact of SMG on the different stages of the adult neurogenesis process in male Long Evans rats experiencing HU. Moreover, we evaluated the role of physical exercise as a countermeasure to protect or limit the effects of SMG on adult neurogenesis. Physical exercise is indeed commonly used to combat the deleterious physiological effects of microgravity exposure in astronauts during spaceflights^38,39^. In addition, several studies demonstrated the significant positive impact of physical exercise on adult neurogenesis^40,41^. In this study, we found that SMG impaired newborn cell proliferation in the DG only after 7 days of exposure. SMG also decreased the short-term (7 days), but not long-term (21 days) survival of newborn cells in both the SVZ/OB and the DG. Physical exercise was able to reverse the impact of SMG on newborn survival observed in these two areas. At the molecular level, RTqPCR and RNA-seq analyses revealed a transient change in the expression of several genes involved in neurogenesis. These findings suggest that adult neurogenesis is transiently impaired by SMG and identify physical exercise as an effective countermeasure to limit the impact of SMG on new neurons generated throughout adulthood.

## Results

### Validation of the HU model

**Fig. 1:**
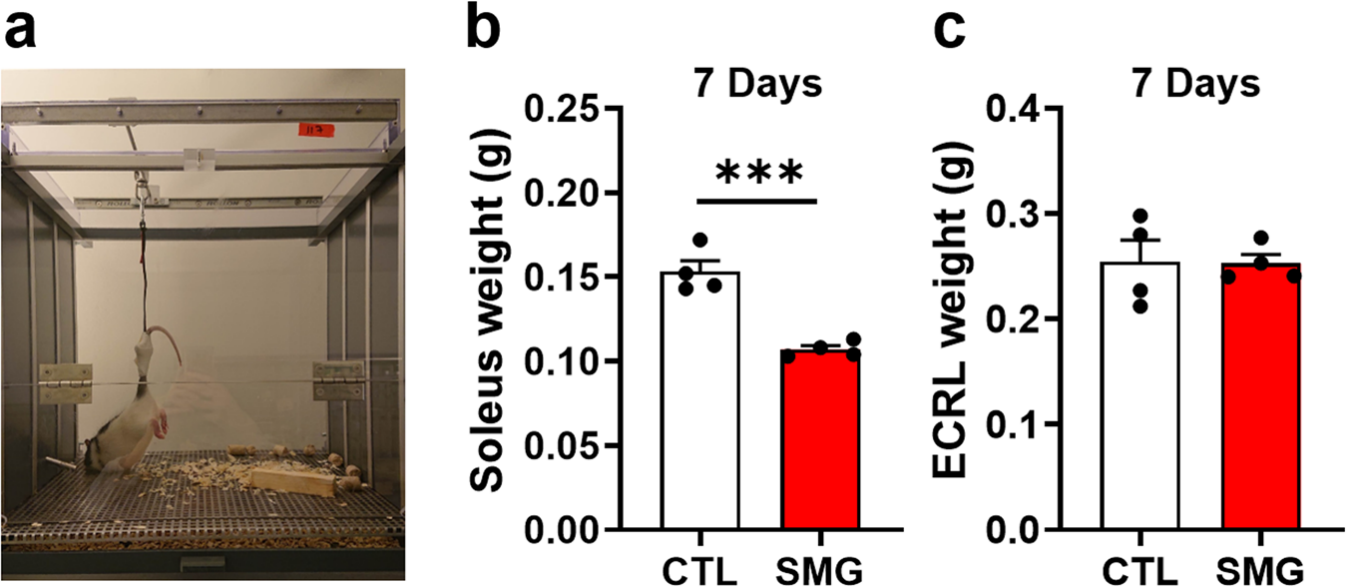
Validation of HU model. **a**. Picture of the HU model. **b** and **c**. Effects of SMG on the weight of the soleus (**b**) and extensor carpi radialis longus (ECRL) (**c**) muscles in CTL (white, *n* = 4) and SMG (red, *n* = 4) rats. All data are presented as mean ± SEM. *** *p* < 0.005.

In this study, the effects of microgravity were mimicked by submitting male Long Evans rats to the HU model (Fig. 1a). To assess its efficiency in our experimental conditions, we weighed the soleus muscle which controls the hindlimbs and known to be impacted by microgravity in astronauts and in the HU model^42^. The extensor carpi radialis longus (ECRL) muscle in the forward limbs, whose weight is not impacted by microgravity in astronauts and in the HU model, served as control. As expected, 7 days of SMG exposure significantly decreased the weight of the soleus muscle (Fig. 1b; Unpaired t-test, t_6_ = 6.57, *p* < 0.001), but not the weight of the ECRL muscle (Fig. 1c; Unpaired t-test, t_6_ = 0.07, *p* = 0.95). The same pattern of effects was observed when the weight of these two muscles was expressed as a function of the total weight of the rats (Supplementary Fig. 1c). Thus, these results validate the effects of HU in our experimental context.

### Simulated microgravity alters the weight gain in rats injected with adult neurogenesis marker

**Fig. 2:**
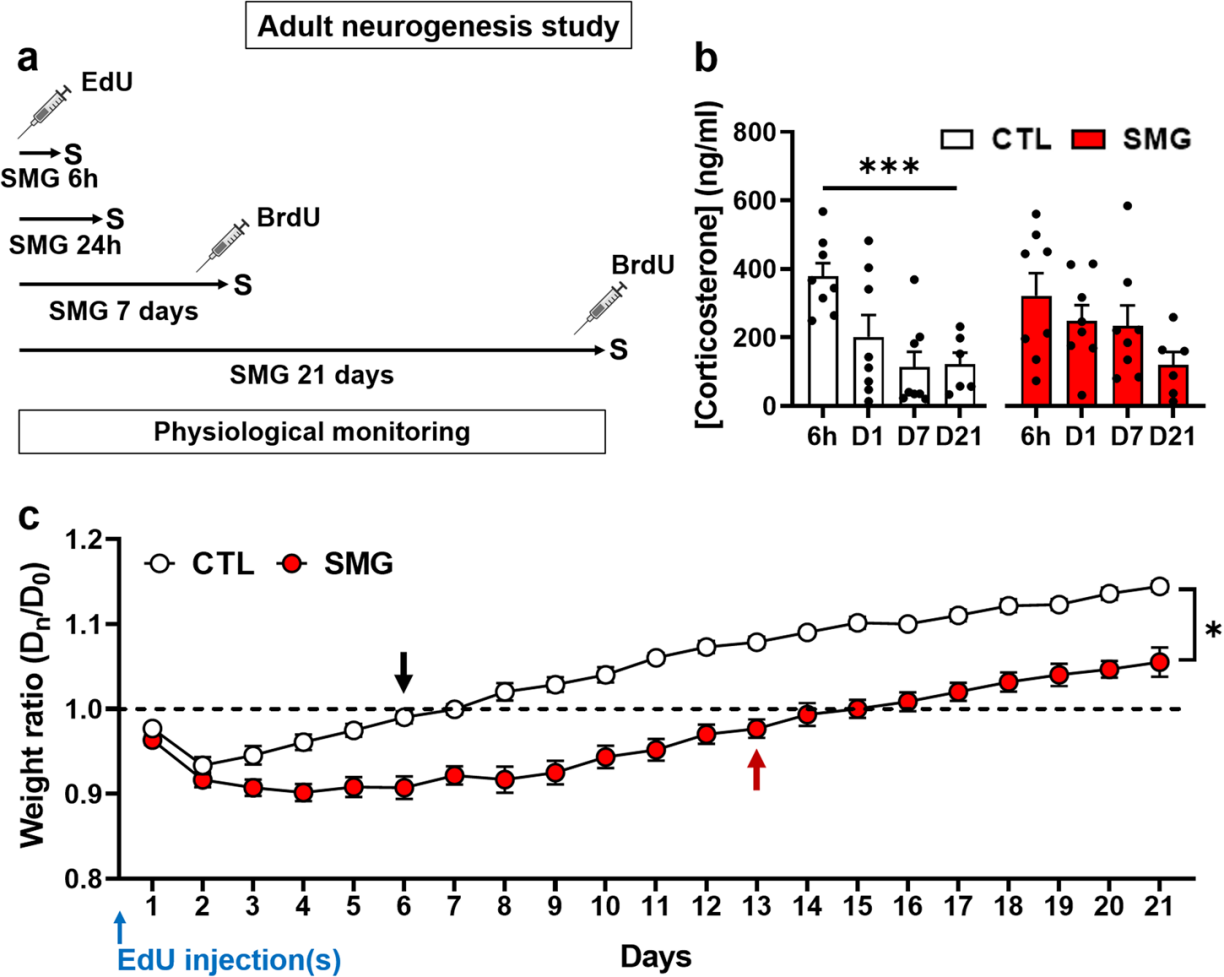
SMG caused a delay in the weight gain of rats injected with the adult neurogenesis marker. **a** Experimental design. EdU was injected just before hindlimb suspension. Rats were exposed to SMG during 6 h, 24 h, 7 days or 21 days. Rats exposed to 7 or 21 days were additionally injected with BrdU 24 h before sacrifice (S). The physiological parameters of the animals were monitored daily for the entire duration of the protocol. **b** Corticosterone concentration in CTL (white) and SMG (red) rats. **c** Body weight of the CTL (white) and SMG (red) rats presented as a ratio relative to their weight at the day of Edu injections. The black and red arrows indicate a return of CTL and SMG rats to their starting weight, respectively. All data are presented as mean ± SEM. *p < 0.05, ***p < 0.005.

The physiological parameters of the rats were carefully monitored during the whole period of SMG exposure. To study adult neurogenesis, rats were injected with the cell birth marker EdU (Day 0) and immediately exposed to hindlimb suspension (SMG) or kept in control condition (CTL) for different time periods ranging from 6 h to 21 days (Fig. 2a). EdU injections induced a stress in both CTL and SMG rats as revealed by a high concentration of corticosterone in plasma 6 h after EdU administration (Fig. 2b). No difference was observed between CTL and SMG rats (two-way ANOVA, SMG effect F(1, 52) = 0.54, *p* = 0.47). Moreover, we observed a significant time effect (two-way ANOVA, time effect F(3, 52) = 6.89, *p* < 0.001). However, corticosterone concentration significantly decreased over time in CTL (white bars; one-way ANOVA F(3, 26) = 6.59, *p* = 0.002) but not in SMG rats (red bars; one-way ANOVA F(3, 26) = 2.01, *p* = 0.14), suggesting that the injection-related stress was potentiated in the SMG condition.

The body weight of CTL rats was decreased compared to their starting weight after EdU administration during 5 days (Fig. 2c, white circles; one-sample t-test, D1 to D5 *p* < 0.005). These rats required 6 days to return to their starting body weight (black arrow; one-sample t-test, D6 to D8 *p* > 0.1) and 9 days to significantly gain weight relative to D0 (one-sample t-test, D9 to D21 *p* < 0.01). The body weight of rats exposed to SMG was also decreased by the EdU administration (Fig. 2c, red circles; one-sample t-test, D1 to D7 *p* < 0.0001, D8 to D11 *p* < 0.01, D12 *p* = 0.045). However, it took them 13 days to return to their starting body weight (red arrow; one-sample t-test, D13 *p* = 0.08, D14 to D17 *p* > 0.1) and 18 days to significantly increase their body weight (one-sample t-test, D18 to D21 *p* < 0.05). A significant difference was observed between CTL and SMG rats from D4 to the end of the experiment (two-way repeated measures ANOVA, mixed-effects model, SMG effect F(1, 42) = 60.91, *p* < 0.001; Bonferroni multiple comparisons test, D1 to D3 *p* > 0.2, D4 to D21 *p* < 0.05). A simple linear regression over 21 days showed that weight gain differed between CTL and SMG rats (F(1, 396) = 41.98, *p* < 0.0001). A more detailed analysis revealed that this difference was due to the first 4 days of exposure. Indeed, from D5, the simple linear regression showed no difference between CTL and SMG rats (F(1, 236) = 0.06, *p* = 0.82), indicating that after an initial phase of body weight stagnation, weight gain in SMG and CTL rats was similar.

The EdU injection effect on the body weight was in part due to a decrease in food consumption observed during the first 4 days in CTL and SMG rats compared with the food consumption at D0 recorded before EdU injection(s) (Supplementary Fig. 1a; two-way repeated measures ANOVA, mixed-effect model, time effect F(3.92, 67.09) = 34.09, *p* < 0.0001; Dunnett multiple comparisons test *vs* D0, CTL: D1 to D4 *p* < 0.01, SMG: D1 to D4 *p* < 0.001). Then, food consumption was similar to the initial consumption in CTL rats (D5 to D21 *p* > 0.1) but more variable in SMG rats (D5 to D7 *p* > 0.05, D8 *p* = 0.044, D9 to D19 *p* > 0.1, D20 *p* = 0.027, D21 *p* = 0.99). However, no difference was observed between CTL and SMG rats at any time (two-way repeated measures ANOVA, mixed-effects model, SMG effect F(1, 43) = 5.18, *p* = 0.028; Bonferroni multiple comparisons test, D1 to D21 *p* > 0.25). This result indicates that despite SMG rats consuming the same amount of food as CTL rats, their weight gain decreased. Water consumption was also slightly altered the first day following the EdU injection(s) (Supplementary Fig. 1b; two-way repeated measures ANOVA, mixed-effect model, time effect F(5.57, 92.76) = 7.26, *p* < 0.0001; Dunnett multiple comparisons test *vs* D0, CTL: D1 *p* = 0.0009, D2 to D21 *p* > 0.15; SMG: D1 *p* < 0.0001; D2 to D21 *p* > 0.15). As for food consumption, no difference was observed between CTL and SMG rats (two-way repeated measures ANOVA, mixed-effects model, SMG effect F(1, 43) = 0.022, *p* = 0.88). No effect of SMG was observed in rat daily temperature (Supplementary Fig. 1c; two-way repeated measures ANOVA, mixed-effect model, time effect F(1, 43) = 2.32, *p* = 0.14) or in glycemia measured at D0, D1, D7, D14 and D21 (Supplementary Fig. 1d; two-way repeated measures ANOVA, mixed-effect model, time effect F(1, 43) = 0.08, *p* = 0.79). Both temperature and glycemia measures were within physiological values (green area).

Overall, SMG potentiated the stressful effect of the EdU injection(s) notably with an effect on the rats’ body weight.

### Simulated microgravity transiently alters the proliferation of newborn hippocampal cells

**Fig. 3:**
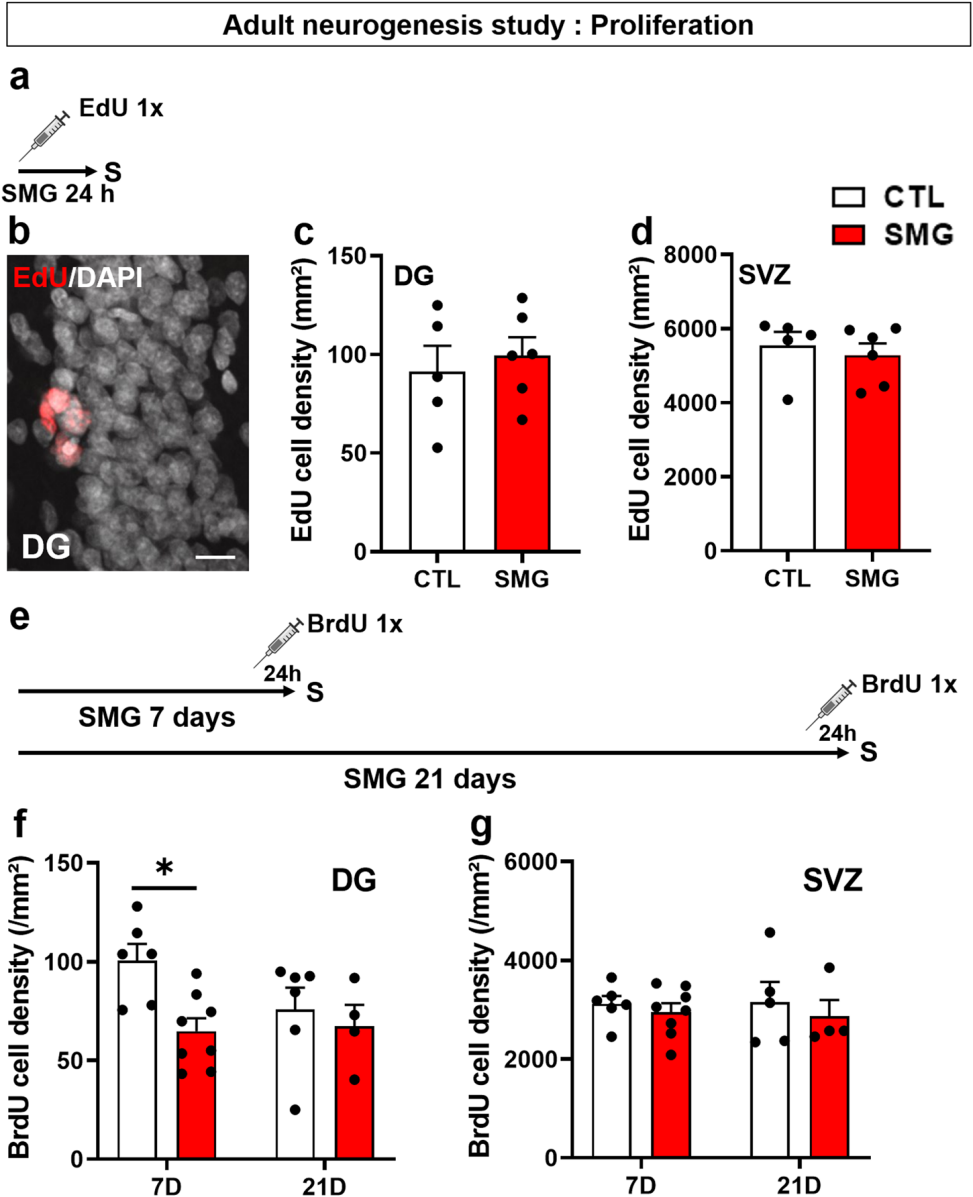
SMG impaired newborn cell proliferation after 7 days of exposure only in the DG. **a** Experimental design. Rats were injected with EdU and exposed to SMG during 24 h. **b** Example of EdU+ cells (in red) in the DG of the hippocampus. Scale bar: 10 µm. **c** Density of EdU cells per mm² in the DG of CTL (white, *n* = 5) and SMG (red, *n* = 6) rats. **d** Density of EdU cells per mm² in the SVZ of CTL (white, *n* = 5) and SMG (red, *n* = 6) rats. **e** Experimental design. Rats were exposed to SMG during 7 or 21 days and injected with BrdU 24 h before the end of the protocol. **f** Density of BrdU cells per mm² in the DG of CTL (white, 7D: *n* = 6; 21D: *n* = 6) and SMG (red, 7D: *n* = 8; 21D: *n* = 4) rats. **g** Density of BrdU cells per mm² in the SVZ of CTL (white, 7D: *n* = 6; 21D: *n* = 5) and SMG (red, 7D: *n* = 8; 21D: *n* = 4) rats. All data are presented as mean ± SEM. * *p* < 0.05.

We evaluated the impact of SMG on newborn cell proliferation in the DG and SVZ of rats injected with EdU and then immediately exposed to SMG during 24 h (Fig. 3a, b). SMG did not affect EdU cell density in the DG (Fig. 3c; Unpaired t-test, t_9_ = 0.52, *p* = 0.61) or in the SVZ (Fig. 3d; Mann–Whitney test, *p* = 0.54). Similar results were observed after 6 h of SMG exposure (Supplementary Fig. 2a–c; DG: unpaired t-test, t_9_ = 0.37, *p* = 0.72; SVZ: unpaired t-test, t_9_ = 1.59, *p* = 0.15). As expected, we observed the well-known time-dependent increase in newborn cell proliferation in the DG between 6 and 24 h (Supplementary Fig. 2b; two-way ANOVA, time effect F(1, 18) = 8.60, p = 0.009). Moreover, the expression of GFAP (Supplementary Fig. 2d) and Sox2 (Supplementary Fig. 2e), known to be expressed in progenitors cells and to be involved in their maintenance and proliferation in the neurogenic niche^43,44^, were not modified by the SMG (Supplementary Fig. 2f, g; three-way ANOVA, SMG effect, DG: F(1, 36) = 0.06, *p* = 0.8; SVZ: F(1, 36) = 0.6, *p* = 0.45). These results indicate that short SMG exposure did not disturb proliferation of the newborn cells in the neurogenic niche.

Because the effects of microgravity can take time to develop in both humans and animals, we next explored if longer exposure to SMG could impair the proliferation phase of adult neurogenesis. To this end, we exposed rats to SMG or control condition during 7 or 21 days. BrdU was injected 24 h before the end of the hindlimb suspension to specifically tag the proliferation phase (Fig. 3e). A significant decrease in BrdU cell density was observed after 7 days of SMG exposure in the DG (Fig. 3f; two-way ANOVA, SMG effect F(1, 20) = 5.76, *p* = 0.026; Bonferroni multiple comparisons test *p* = 0.013), but not after 21 days (Bonferroni multiple comparisons test *p* > 0.99). No effect was observed in the SVZ (Fig. 3g; two-way ANOVA, SMG effect F(1, 19) = 0.75, *p* = 0.4). These results indicate that longer exposure to SMG transiently disturbs hippocampal newborn cell proliferation.

### Simulated microgravity transiently alters newborn cell survival

**Fig. 4:**
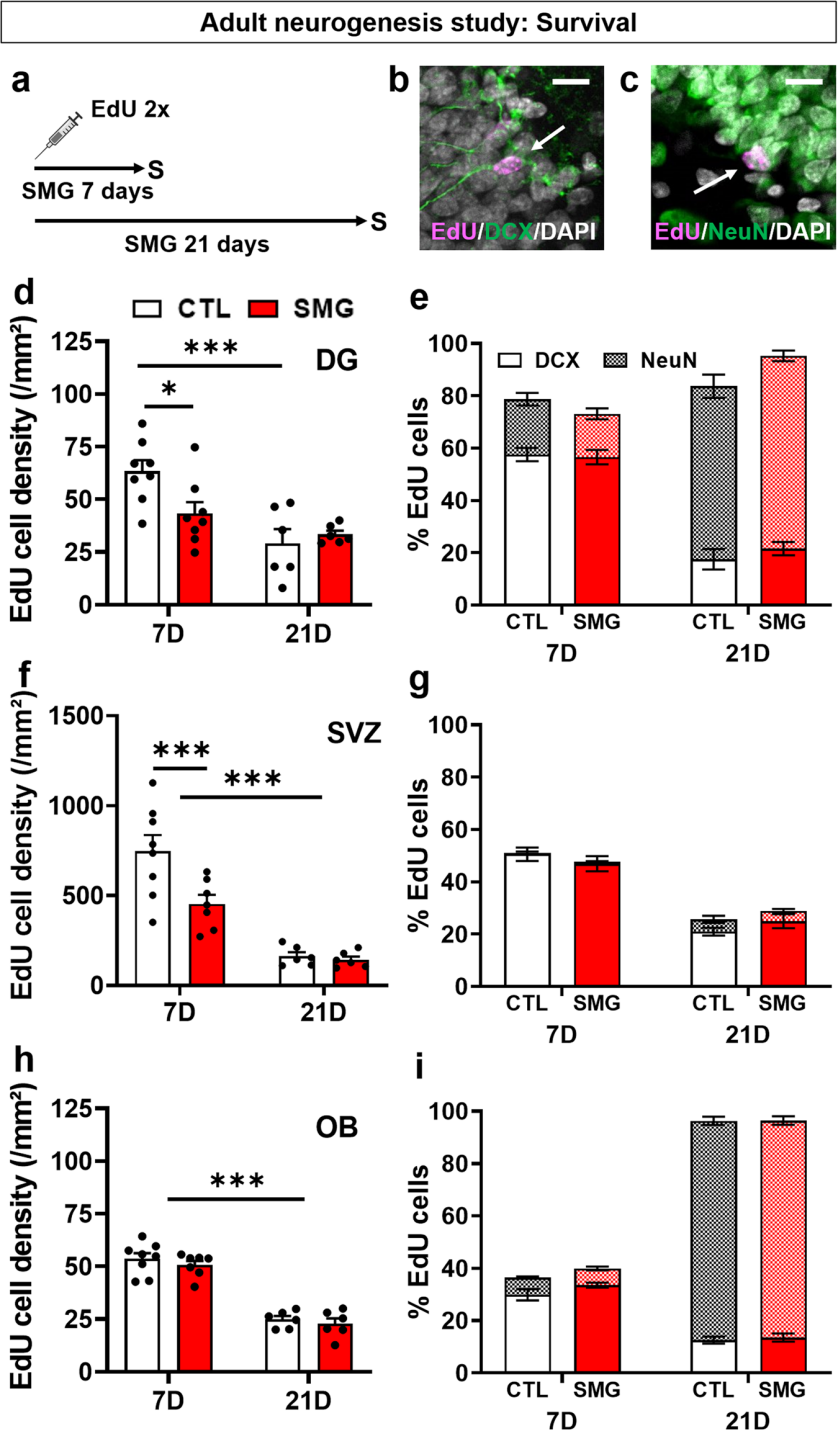
SMG resulted in impaired newborn cell survival after 7 days of exposure in the DG and SVZ. **a** Experimental design. Rats were injected with EdU and exposed to SMG during 7 or 21 days. **b** Example of EdU+ cell expressing DCX in the DG of the hippocampus. Scale bar: 10 µm. **c** Example of EdU+ cell expressing NeuN in the DG of the hippocampus. Scale bar: 10 µm. **d** Density of EdU cells per mm² in the DG of CTL (white, 7D: *n* = 8; 21D: *n* = 6) and SMG (red, 7D: *n* = 8; 21D: *n* = 6) rats. **e** Percentage of EdU cells expressing DCX or NeuN in the DG of CTL (white) and SMG (red) rats. **f** Density of EdU cells per mm² in the SVZ of CTL (white, 7D: *n* = 8; 21D: *n* = 6) and SMG (red, 7D: *n* = 7; 21D: *n* = 6) rats. **g** Percentage of EdU cells expressing DCX or NeuN in the SVZ of CTL (white) and SMG (red) rats. **h** Density of EdU cells per mm² in the OB of CTL (white, 7D: *n* = 8; 21D: *n* = 6) and SMG (red, 7D: *n* = 7; 21D: *n* = 6) rats. **I** Percentage of EdU cells expressing DCX or NeuN in the OB of CTL (white) and SMG (red) rats. All data are presented as mean ± SEM. *p < 0.05, **p < 0.01, ***p < 0.005.

We next explored the effect of SMG on newborn cell survival in the DG, SVZ and OB. Rats were injected with EdU and then immediately exposed to SMG during 7 or 21 days (Fig. 4a). As expected, we observed in CTL rats the well-known time-dependent decrease in newborn cell survival between 7 and 21 days in the 3 structures (DG: Fig. 4d, two-way ANOVA, time effect F(1, 24) = 16.9, *p* = 0.0004; SVZ: Fig. 4f, two-way ANOVA, time effect F(1, 23) = 51.96, *p* < 0.001; OB: Fig. 4h, two-way ANOVA, time effect F(1, 23) = 142.1, *p* < 0.001). In the DG, EdU cell density was significantly decreased after the 7-day SMG exposure period (Fig. 4d; two-way ANOVA, SMG effect F(1, 24) = 2.17, *p* = 0.15, interaction F(1, 24) = 5.18, *p* = 0.032; Bonferroni multiple comparisons test *p* = 0.017) but not after 21 days (*p* > 0.99). A similar result was observed in the SVZ (Fig. 4f; two-way ANOVA, SMG effect F(1, 23) = 6.4, *p* = 0.019; Bonferroni multiple comparisons tests: 7d *p* = 0.003; 21D *p* > 0.99). In addition, social isolation was not a confounding factor (Supplementary Fig. 3). In the OB, no difference was observed between CTL and SMG (Fig. 4h; two-way ANOVA, SMG effect F(1, 23) = 1.17, *p* = 0.29).

In CTL rats, we observed a decrease in DCX expression over time associated with an increase in NeuN expression in the DG (Fig. 4e, three-way ANOVA with repeated measures, time effect F(1, 23) = 20.1, *p* < 0.001; Bonferroni test 7D *vs* 21D: DCX *p* < 0.001, NeuN *p* < 0.001) and in OB (Fig. 4i, three-way ANOVA with repeated measures, time effect F(1, 24) = 1354, *p* < 0.001; Bonferroni test 7D *vs* 21D: DCX *p* < 0.001, NeuN *p* < 0.001). In SVZ, we observed a decrease of DCX expression over time (Fig. 4g, three-way ANOVA with repeated measures, time effect F(1, 24) = 74.04, *p* < 0.001; Bonferroni test 7D *vs* 21D *p* < 0.001). These results indicate that the neurons matured properly in the different structures analyzed. SMG did not alter the expression of DCX and/or NeuN in newborn cells in the DG (Fig. 4e; three-way ANOVA with repeated measures, SMG effect F(1, 23) = 0.85, *p* = 0.37), the SVZ (Fig. 4g; three-way ANOVA with repeated measures, SMG effect F(1, 24) = 0.005, *p* = 0.95) and in the OB (Fig. 4i; three-way ANOVA with repeated measures, SMG effect F(1, 24) = 1.34, *p* = 0.26). These results indicate that exposure to SMG transiently disturb newborn cell survival in DG and SVZ, without affecting the maturation of these cells.

### Physical exercise facilitates physical recovery after injections of the adult neurogenesis marker

**Fig. 5:**
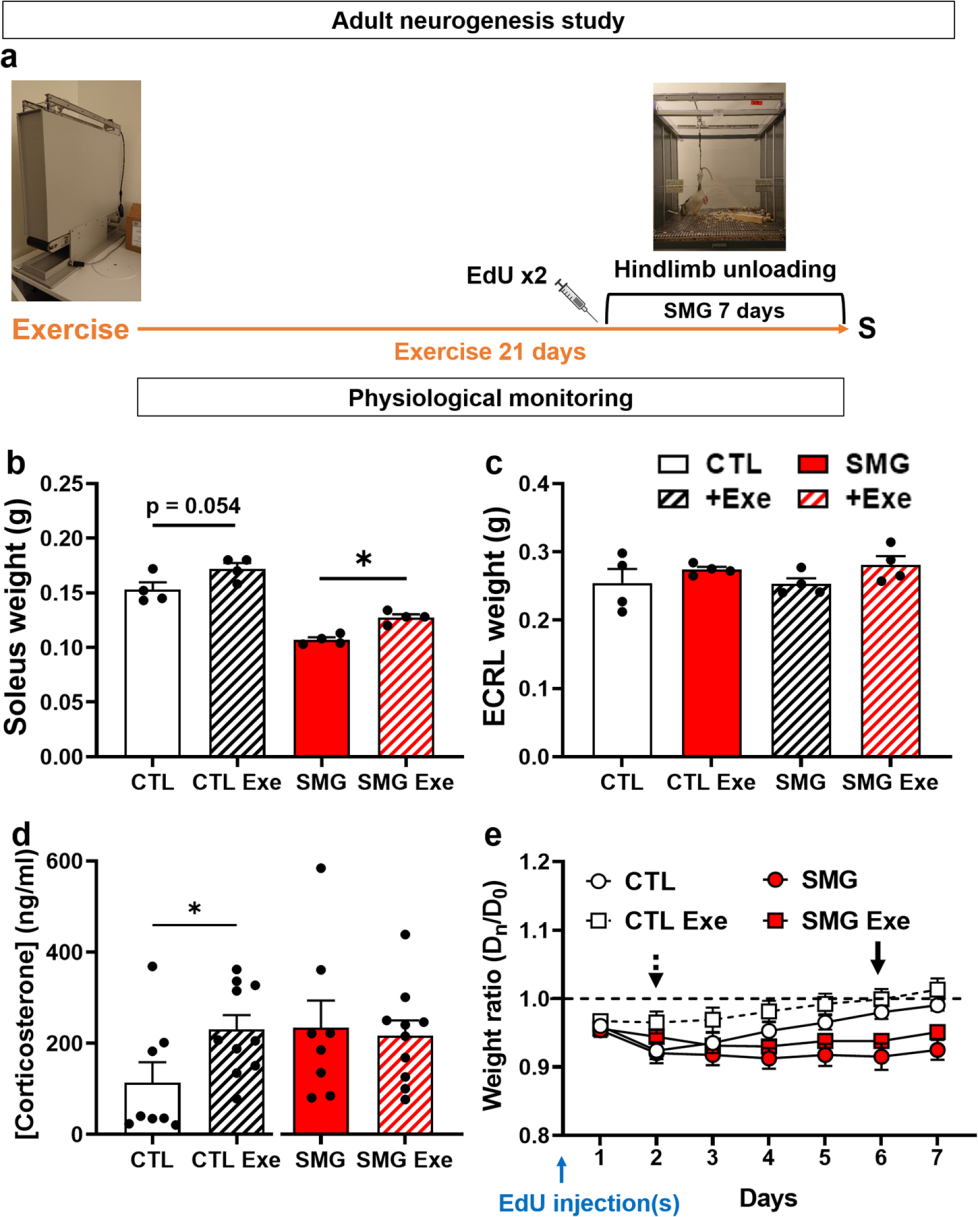
Exercise improves weight recovery in rats after injections with the adult neurogenesis marker. **a** Experimental design. Rats were exposed to physical exercise during 3 weeks. After 2 weeks, rats were injected with EdU and exposed to SMG for 7 days. The physiological parameters of the animals were monitored daily. **b** Weight of the soleus muscle in CTL (white, *n* = 4) and SMG (red, *n* = 4) rats exposed or not to physical exercise. **c** Weight of the extensor carpi radialis longus (ECRL) muscle in CTL (white, *n* = 4) and SMG (red, *n* = 4) rats exposed or not to physical exercise. **d** Corticosterone concentration in CTL (white, CTL: *n* = 8; CTL Exe: *n* = 10) and SMG (red, SMG: *n* = 8; SMG Exe: *n* = 10) rats exposed or not to physical exercise. **e** Weight of the CTL (white, CTL: *n* = 8; CTL Exe: *n* = 10) and SMG (red, SMG: *n* = 8; SMG Exe: *n* = 10) rats exposed or not to physical exercise presented as a ratio compared with the weight at D0. Black arrow marks the point where the CTL rats return to their starting weight. The black dotted arrow marks the point where the CTL rats exposed to physical exercise return to their starting weight. All data are presented as mean ± SEM. *p < 0.05.

During spaceflights, astronauts are submitted to a strict physical exercise routine to limit muscle loss in the forward limbs and the effects of microgravity on the cardiovascular system. We explored the impact of physical exercise on adult neurogenesis during SMG exposure. For this, rats were exposed to physical exercise in a treadmill during 2 weeks prior to the SMG exposure and 1 week during the SMG exposure (Fig. 5a). Rats were injected with EdU just before the SMG exposure (Fig. 5a). No difference was observed in the distance covered by the rats during the 3 weeks of exposure to physical exercise between the CTL and the SMG conditions (CTL: 461 ± 9 m/rat/day *vs* SMG: 465 ± 5 m/rat/day; unpaired t-test, t_18_ = 0.44, *p* = 0.67) or during the last week, i.e., during SMG exposure (CTL: 477 ± 23 m/rat/day *vs* SMG: 471 ± 15 m/rat/day, unpaired t-test, t_18_ = 0.23, *p* = 0.82). This indicates that SMG did not impair the motor skills of the rats to perform physical exercise on the treadmill.

Physical exercise increased the weight of the soleus muscle in CTL and SMG rats (Fig. 5b; two-way ANOVA, exercise effect F(1, 12) = 18.45, *p* = 0.001; Tukey multiple comparisons test, CTL *vs* CTL Exe *p* = 0.054, SMG *vs* SMG Exe *p* = 0.036). No weight change was observed for the ECRL muscle (Fig. 5c; two-way ANOVA, exercise effect F(1, 12) = 3.39, *p* = 0.09). The same result was observed when the weight of the muscles was expressed as the total body weight of the rats (Supplementary Fig. 4e, f). These results indicate that physical exercise is efficient in limiting muscle loss in SMG rats. Moreover, physical exercise did not change the stress level in SMG rats (Fig. 5d, red; unpaired t-test, t_16_ = 0.28, *p* = 0.79), but a significant effect was observed in CTL (Fig. 5d, white; Mann–Whitney test, U_16_ = 17, *p* = 0.043).

We previously reported that the body weight of the CTL rats was decreased after EdU injections and it took them 6 days to return to their starting weight (Fig. 2d and Fig. 5e, black arrow). Physical exercise reduced the recovery time after EdU injections by 4 days (Fig. 5e, black dotted arrow; CTL: one-sample t-test, D1 to D5 *p* < 0.05, D6 *p* = 0.08, D7 *p* = 0.24; CTL Exe: one-sample t-test, D1 *p* = 0.005, D2 *p* = 0.058, D3 to D7 *p* > 0.1). This result indicates that physical exercise facilitates weight recovery in CTL rats after EdU injections. In SMG rats, we did not observe a beneficial effect of the physical exercise on weight recovery time during the 7 days of observation. No difference was observed between rats exposed to exercise or not in both conditions (three-way ANOVA, exercise effect F(1, 32) = 3.58, *p* = 0.07, interaction SMG x Exercise F(1, 32) = 0.11, *p* = 0.75). Simple linear regressions showed no difference induced by physical exercise in CTL (F(1, 122) = 0.01, *p* = 0.91) or SMG rats (F(1, 115) = 0.24, *p* = 0.63). As previously observed, a significant difference between CTL and SMG rats without exercise was observed (F(1, 108) = 6.28, *p* = 0.014), but also with exercise (F(1, 129) = 4.9, *p* = 0.028). These results indicate that the gain weight was different in SMG rats compared with CTL rats during the first week of exposure to SMG.

Food consumption was reduced during 3 days after EdU injections in CTL rats (Supplementary Fig. 4a; two-way ANOVA, time effect F(3.533, 49.46) = 30.68, *p* < 0.0001; Dunnett multiple comparisons test *vs* D0: D1 to D3 *p* < 0.01, D4 to D7 *p* > 0.2) and 2 days in SMG rats (Dunnett multiple comparisons test *vs* D0: D1 to D2 *p* < 0.05, D3 to D7 *p* > 0.1). Physical exercise decreased the recovery time in CTL rats by 2 days (Dunnett multiple comparisons test *vs* D0, D1 *p* < 0.001, D2 to D7 *p* > 0.1), but not in SMG rats (Dunnett multiple comparisons test *vs* D0: D1 to D3 *p* < 0.01, D4 to D7 *p* > 0.2). No difference was observed between rats exposed to exercise or not in both conditions (three-way ANOVA, exercise effect F(1, 32) = 4.06, *p* = 0.052; Tukey multiple comparisons test *p* > 0.6).

Water consumption decreased the day after the EdU injections in CTL (Supplementary Fig. 4b; two-way ANOVA, time effect F(5.189, 72.64) = 11.72, *p* < 0.001; Dunnett multiple comparisons tests *vs* D0: D1 *p* = 0.005, D2 to D7 *p* > 0.5) and SMG rats (D1 *p* = 0.005, D2 to D7 *p* > 0.35). With physical exercise, CTL rats did not show decrease in water consumption (Dunnett multiple comparisons tests *vs* D0, D1 to D7 *p* > 0.05). In contrast, in SMG rats, physical exercise did not change the water consumption’ decrease (Dunnett multiple comparisons tests *vs* D0, D1 *p* = 0.052, D2 to D7 *p* > 0.1). Moreover, no difference was observed between rats exposed to exercise or not in both gravity conditions (three-way ANOVA, exercise effect F(1, 32) = 1.04, *p* = 0.31).

Physical exercise increased the body temperature in both CTL and SMG rats (Supplementary Fig. 4c; three-way ANOVA, exercise effect F(1, 32) = 56.43, *p* < 0.001). Physical exercise also increased the glycemia (Supplementary Fig. 4d right; D0, two-way ANOVA, exercise effect F(1, 32) = 27.06, *p* < 0.001). However, this effect was only visible at the end of the exercise period while no effect was observed at D7 (sacrifice day without exercise, Supplementary Fig. 4d left, two-way ANOVA, exercise effect F(1, 32) = 4.04, *p* = 0.053). In all cases, temperature and glycemia measures stayed within physiological values (green area).

### Physical exercise restores the survival of newborn cells in DG and SVZ

**Fig. 6:**
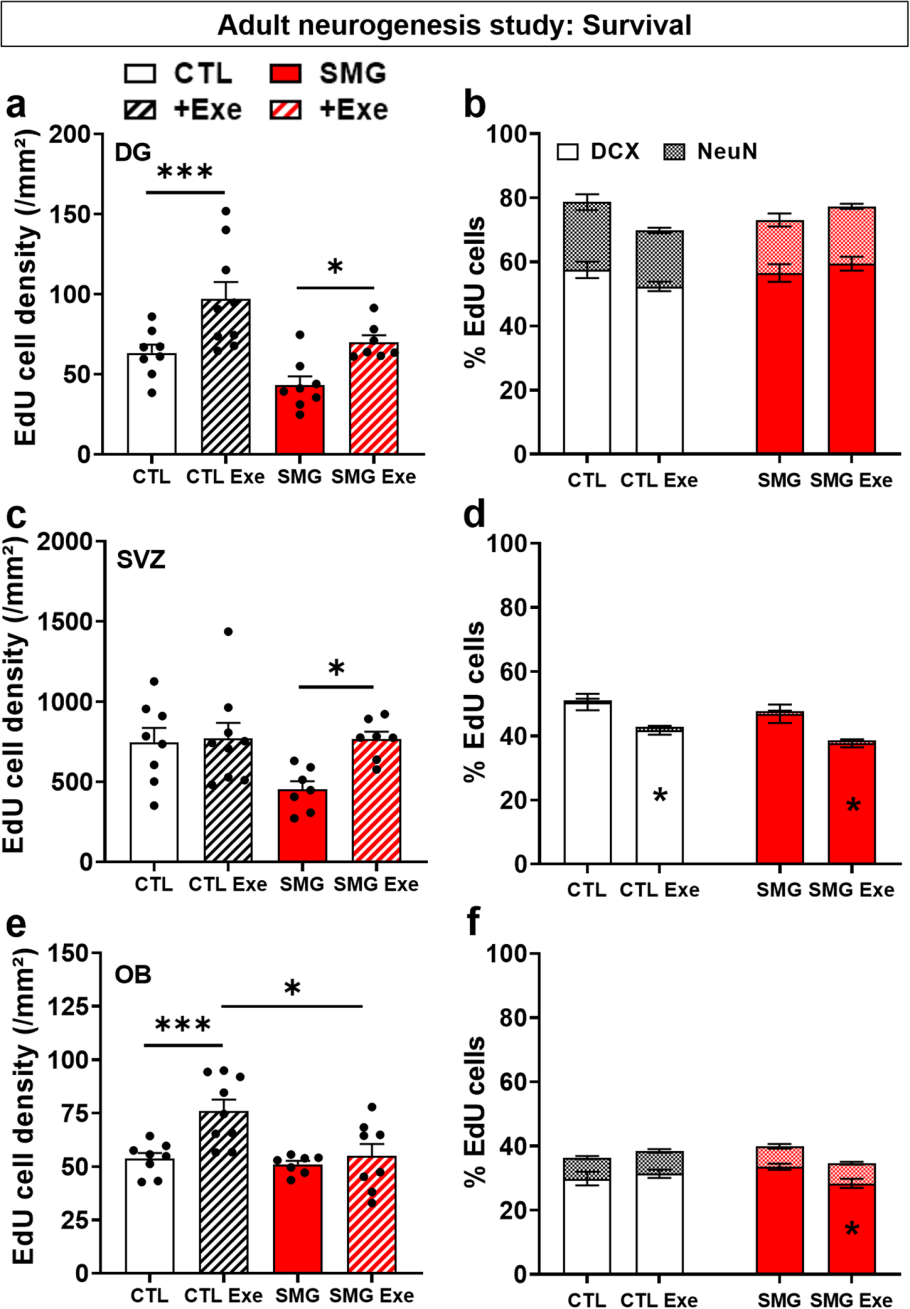
Physical exercise restored the survival of newborn cells in the DG and SVZ. **a** Density of EdU cells per mm² in the DG of CTL (white, CTL: *n* = 8; CTL Exe: *n* = 9) and SMG (red, SMG: *n* = 8; SMG Exe: *n* = 7) rats exposed or not to physical exercise. **b** Percentage of EdU cells expressing DCX or NeuN in the DG of CTL (white) and SMG (red) rats. **c** Density of EdU cells per mm² in the SVZ of CTL (white, CTL: *n* = 8; CTL Exe: *n* = 9) and SMG (red, SMG: *n* = 8; SMG Exe: *n* = 7) rats exposed or not to physical exercise. **d** Percentage of EdU cells expressing DCX or NeuN in the SVZ of CTL (white) and SMG (red) rats. **e** Density of EdU cells per mm² in the OB of CTL (white, CTL: *n* = 8; CTL Exe: *n* = 9) and SMG (red, SMG: *n* = 8; SMG Exe: *n* = 7) rats exposed or not to physical exercise. **f** Percentage of EdU cells expressing DCX or NeuN in the OB of CTL (white) and SMG (red) rats. All data are presented as mean ± SEM. *p < 0.05, ***p < 0.005.

As expected, physical exercise increased the EdU density in CTL rats in the DG (Fig. 6a, black; two-way ANOVA, exercise effect F(1, 28) = 16.73, *p* < 0.001; Bonferroni multiple comparisons test *p* = 0.005) and in the OB (Fig. 6e, black; two-way ANOVA, exercise effect F(1, 28) = 9.05, *p* = 0.006; Bonferroni multiple comparisons test *p* = 0.002). No effect of physical exercise was observed in the SVZ (Fig. 6c, black; two-way ANOVA, exercise effect F(1, 27) = 4.36, *p* = 0.046; Bonferroni multiple comparisons test *p* > 0.99). These results indicate that our physical exercise paradigm was able to enhance newborn cells’ survival.

Physical exercise increased the density of EdU cells in SMG rats in the DG (Fig. 6a, Bonferroni multiple comparisons test, *p* = 0.038) and SVZ (Fig. 6c, Bonferroni multiple comparisons test, *p* = 0.028). No effect of physical exercise was observed in the OB of SMG rats (Fig. 6e, Bonferroni multiple comparisons test, *p* > 0.99). No difference was observed between CTL and SMG Exe rats in the DG or SVZ (Fig. 6a, c, Bonferroni multiple comparisons test, *p* > 0.99), showing that 3 weeks of physical exercise was enough to restore survival of newborn cells to a level similar to that of CTL rats. In contrast, a significant difference was observed between CTL Exe and SMG Exe rats in the OB (Fig. 6e, Bonferroni multiple comparisons test, *p* = 0.012), indicating that SMG inhibits the positive effect of physical exercise observed on the survival of the newborn cells in the OB.

No effect of exercise was observed in DCX and NeuN expression in CTL or SMG rats in DG (Fig. 6b, three-way ANOVA repeated measures, Exercise effect F(1, 29) = 0.99, *p* = 0.33). In SVZ, exercise decreased the percentage of DCX cells in both CTL and SMG rats (Fig. 6d, three-way ANOVA repeated measures, Exercise effect F(1, 58) = 16.94, *p* < 0.001; Tukey’s multiple comparisons test, exercise *vs* no exercise, CTL, *p* = 0.002; SMG, *p* = 0.002). No difference was observed in NeuN expression (CTL, *p* > 0.99; SMG, *p* > 0.99). In the OB, we observed no effect of exercise on DCX and NeuN expression in CTL rats (Fig. 6f, three-way ANOVA repeated measures, F(1, 29) = 0,99, *p* = 0.33, interaction exercise x SMG, F(1, 29) = 5.14, *p* = 0.03; Tukey’s multiple comparisons test, exercise *vs* no exercise DCX *p* = 0.98, NeuN *p* > 0.99). However, exercise decreased the percentage of DCX cells in SMG rats (*p* = 0.043).

### Simulated microgravity slightly alters the weight gain in rats in gene expression experiment

**Fig. 7:**
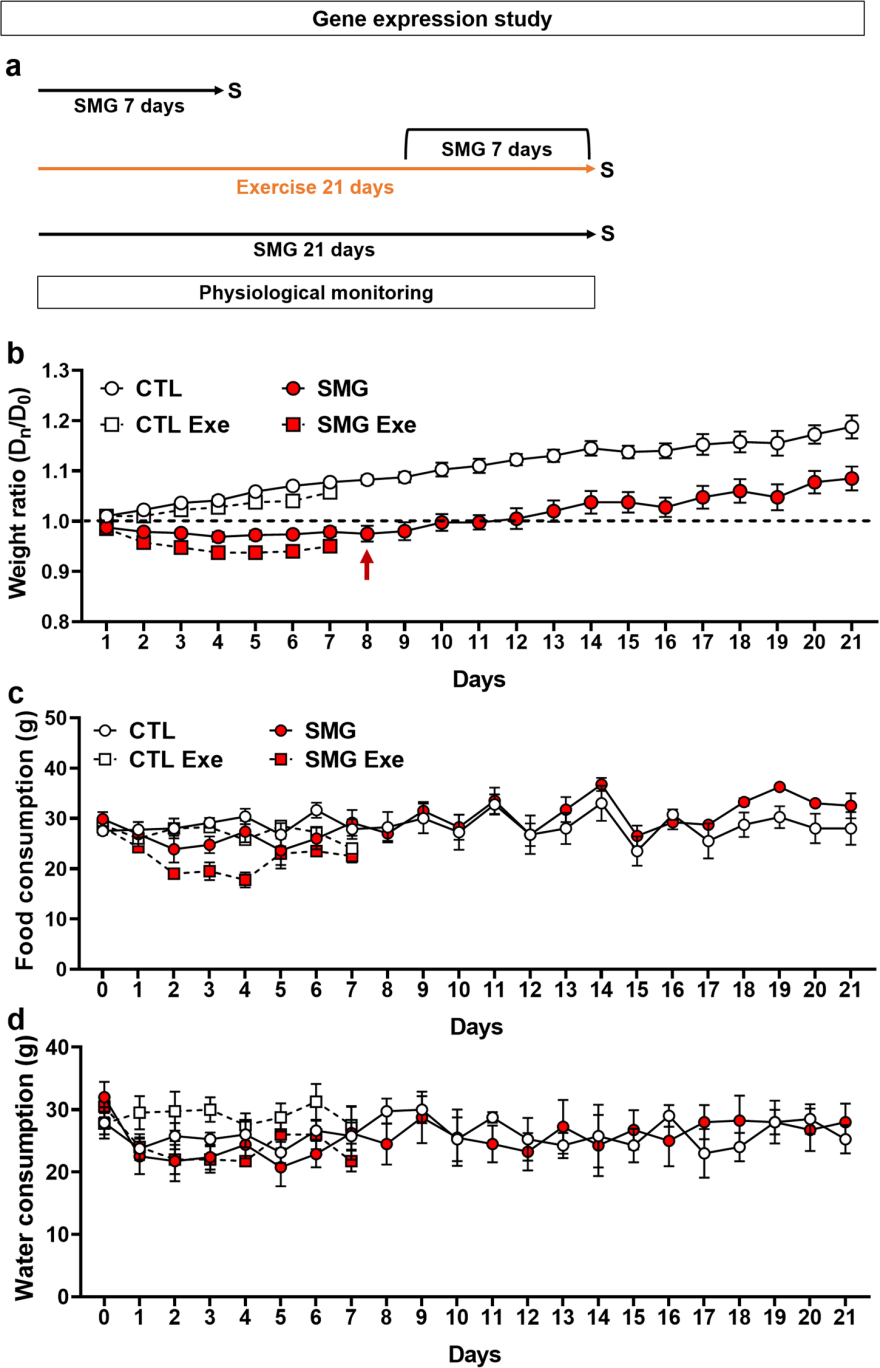
SMG slightly altered the weight gain in rats. **a** Experimental design. Rats were exposed to SMG during 7 or 21 days. Some rats were exposed to physical exercise during 3 weeks. After 2 weeks, rats were exposed to SMG for 7 days. The physiological parameters of the animals were monitored daily. **b** Weight of the CTL (white) and SMG (red) rats exposed (square) or not (circle) to physical exercise presented as a ratio compared with the weight at D0. The red arrow marks the point where the SMG rats return to their starting weight. **c** Food consumption of the CTL (white) and SMG (red) rats exposed (square) or not (circle) to physical exercise. **d** Water consumption of the CTL (white) and SMG (red) rats exposed (square) or not (circle) to physical exercise. All data are presented as mean ± SEM.

In this experiment, dedicated to gene expression analyses, neurogenesis was not assessed. Accordingly, rats were submitted for 7 or 21 days to SMG exposure without EdU/BrdU injections (Fig. 7a). In these conditions, CTL rats did not lose weight and they continuously gained weight from D1 (Fig. 7b, white circle and black arrow; one-sample t-test, D1 to D21 *p* < 0.05). In contrast, SMG rats lost weight (Fig. 7b, red circle) and it took them 8 days to return to their starting weight (Fig. 7b, red arrow; one-sample t-test, D1 to D7 *p* < 0.05; D8 *p* = 0.2) and 12 more days to increase their weight (one-sample t-test, D9 to D19 *p* > 0.05, D20 to D21 *p* < 0.05). A significant difference between CTL and SMG was observed during the 8 first day (two-way repeated measures ANOVA, mixed-effects model, SMG effect F(1, 14) = 88.18, *p* < 0.001; Bonferroni multiple comparisons test, D1 to D8 *p* < 0.05; D9 *p* = 0.08, D10 *p* = 0.06, D11 *p* = 0.03, D12 to D21 *p* > 0.1). The lack of difference between D9 to D21 was mostly due to the low number of animals in both groups (*n* = 4). A simple linear regression on the 21 days showed that weight gain differed between CTL and SMG rats (F(1, 206) = 10.66, *p* = 0.001). A more detailed analysis showed that this difference was due to the first 2 days of exposure. Indeed, from D3, the simple linear regression showed no difference between CTL and SMG rats (F(1, 174) = 3.47, *p* = 0.064), indicating that after an initial phase of weight stagnation, SMG rats gain weight in the same way as CTL rats. Physical exercise had no effect on weight gain in CTL rats (Fig. 7b, white square; two-way ANOVA, exercise effect F(1, 10) = 4.08, *p* = 0.07). Simple linear regression showed no difference between rats with or without exercise (F(1, 80) = 1.22, *p* = 0.27). In SMG rats, physical exercise slightly decreased the weight gain from D3 (Fig. 7b, red square; two-way ANOVA, exercise effect F(1, 10) = 9.08, *p* = 0.013; Bonferroni multiple comparisons test, D1 to D2 *p* > 0.3, D3 *p* = 0.043, D4 *p* = 0.1, D5 *p* = 0.09, D6 *p* = 0.005, D7 *p* = 0.12). However, no difference was observed by simple linear regression between SMG rats with or without exercise (F(1, 73) = 1.88, *p* = 0.18). These results indicate that physical exercise associated with SMG could impair the rat’s body weight, at least during several days. However, this effect was not due to stress while physical exercise did not change the corticosterone level in rats (data not shown; two-way ANOVA, exercise effect F(1, 12) = 0.05, *p* = 0.82).

Food and water consumption were stable throughout the experiment in CTL and SMG rats (Food: Fig. 7c, two-way ANOVA, mixed-effects model, time effect F(3.16, 27.35) = 3.93, *p* = 0.017; Dunnett multiple comparisons test, D1 to D21 *p* > 0.05 in both group; Water: Fig. 7d, two-way ANOVA, mixed-effects model, time effect F(2.02, 17.48) = 2.17, *p* = 0.14). No difference was observed between CTL and SMG rats (Food: Fig. 7c, two-way ANOVA, mixed-effects model, SMG effect F(1, 14) = 0.15, *p* = 0.7; Water: Fig. 7d, two-way ANOVA, mixed-effects model, SMG effect F(1, 14) = 0.03, *p* = 0.86). Physical exercise did not change rats’ food (Fig. 7c; three-way ANOVA, exercise effect F(1, 20) = 3.05, *p* = 0.1) or water consumption (Fig. 7d; three-way ANOVA, exercise effect F(1, 20) = 0.63, *p* = 0.44).

No difference was observed in CTL and SMG rats in temperature (data not shown; two-way ANOVA, mixed-effects model, SMG effect F(1, 14) = 0.51, *p* = 0.49) or glycemia (data not shown; two-way ANOVA, mixed-effects model, SMG effect F(1, 14) = 0.05, *p* = 0.83). As previously observed, physical exercise increased the body temperature (data not shown; three-way ANOVA, exercise effect F(1, 20) = 37.77, *p* < 0.001) and the glycemia (data not shown; D0, two-way ANOVA, exercise effect F(1, 20) = 9.41, *p* = 0.006).

### SMG modulates the expression of genes involved in neurogenic processes

**Fig. 8:**
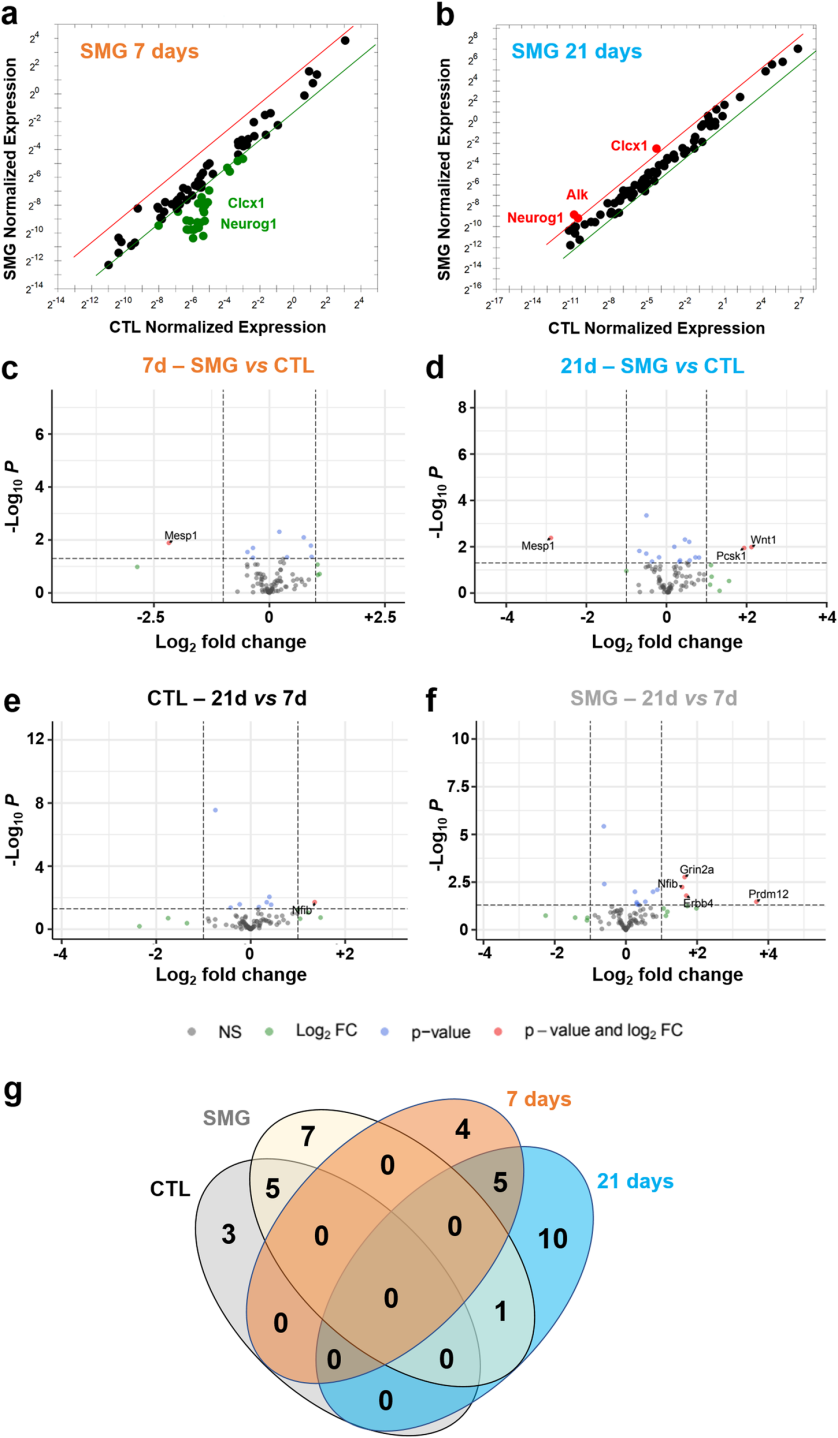
SMG modulates neurogenesis related gene expression in hippocampus. **a**, **b** Comparison of normalized expression of genes of rats exposed to SMG during 7 and 21 days, respectively and revealed by RTqPCR. Comparisons are made with control rats. Dots represent the tested genes with a fold change threshold = 2.5 (red dots on the left of the red line are upregulated and green dots on the right green line are downregulated. Graphs are generated by gene study analyses with CFX software (Bio-Rad). **c**, **d** Volcano plots of expressions of genes of rats exposed to SMG during 7 or 21 days, respectively, revealed by RNAseq. **e** Volcano plot of expressions of genes of CTL rats isolated 21 days compared with a 7-day isolation. **f** Volcano plot of expression of genes of rats exposed to SMG during 21 days compared with a 7-day exposure. In gray (NS) is the group of the non-affected genes, in blue (*p* < 0.05) the expression of gene is significantly affected, in green the fold is change more than twice (|log2FC| > 2) and finally in red are indicated the gene expression significantly and highly changed (*p* < 0.05 and |log2FC| > 2). **g**. Venn’s diagram to segregate affected gene populations according to the experimental conditions.

The regulation of neurogenesis was first investigated by RT-qPCR using a manufactured plate testing 82 genes known to be related to neurogenesis. After 7 days of SMG exposure, the hippocampal expression of 25 genes were decreased with a 2.5-fold change threshold (Fig. 8a and Table 1). After 21 days of SMG exposure, the expression of only 3 genes was increased (Fig. 8b and Table 1). The comparison between 7 and 21 days of SMG exposure indicate that the genes *Clcx1* and *Neurog1*, were regulated in an opposite way: 7 days of exposure decreased their expression while 21 days of exposure increased it. These results suggest that SMG had a transient effect on gene expression involved in neurogenesis in the hippocampus.

**Table 1:** List of genes affected by 7- or 21-day SMG exposure compared to CTL groups by RTqPCR analysis

SMG *vs* CTL 7D (down regulated)		
Gene symbol	Gene ID	Gene Name
Adora1	29290	Adenosine receptor A1
Adora2a	25369	Adenosine receptor A2a
Ascl1	64186	Ashaete-scute homolog 1
BCl2	24224	Apoptosis regulator BCl2
Bmp8a	680931	Bone morphogenetic protein 8a precursor
Cdk5r1	116671	Cyclin dependent kinase 5 activator 1
**Cxcl1**	**81503**	**Growth-regulated alpha protein precursor**
Dll1	84010	Delta like protein 1 precursor
Efnb1	25186	Ephrin-b1 precursor
Ep300	620036	E1A binding protein p300
Erbb2	24337	Receptor tyrosin-protein kinase erbB-2 precursor
Gdnf	25453	Glial cell line-derived neurotrophic factor precursor
Hdac4	363287	Histone deacetylase 4
Hey1	155437	Hary/enhancer-off-split related with Yrpw motif protein 1
Hey2	155430	Hary/enhancer-off-split related with Yrpw motif protein 2
Lif	60584	Leukemia inhibitory factor precursor
**Neurog1**	**29410**	**Neurogenin-1**
Neurog2	295475	Neurogenin-2
Nog	25495	Noggin precursor
Pax3	114502	Paired box 3
Pax6	25509	Paired box protein 6
Pou3f3	192109	Pou domain, class 3, transcription factor 3
Shh	29499	Sonic hedgehog protein precursor
Sox2	499593	Sry-box 2
Sox8	302993	Transcription factor Sox8
**SMG** ***vs*** **CTL 21D (up-regulated)**		
Alk	266802	Alk tyrosin kinase receptor precursor
**Cxcl1**	**81503**	**Growth-regulated alpha protein precursor**
**Neurog1**	**29410**	**Neurogenin-1**

Bold indicates genes regulated in opposite way between 7 and 21 days of SMG.

To complete the analysis of gene expression implicated in adult neurogenesis, we extracted the genes included in the Go-terms “neurogenesis” containing 89 genes from a large RNA-Seq transcriptomic analysis. We analyzed the transcriptomes in 4 rats per experimental groups and compared the results pairwise. We focused only on the genes whose expression was significantly modified (*p* < 0.05) and they were highlighted in the volcano plots when the fold change (FC) was |log2FC| > 2). In SMG rats, the expression of 20 genes involved in neurogenesis were significantly modified (SMG *vs* CTL; Fig. 8c, d and Table 2): 4 were affected after 7 days of SMG exposure (*Ascl1, Celsr1, Prdm8* and *Xrcc5*); 11 were affected after the 21-day SMG exposure period (*Adgrv1, Ercc6, Lft20, Nav1, Nav2, Ndufs2, Pcsk1, Tacc1, Trak1* and *Wnt1*); and 5 were affected identically by both delays of exposure (*Fabp7, Mesp1, Nfix, Smarca4, Tuba1a*). Only *Mesp1* was highly decreased for both SMG durations (|log2FC|> 2). We next compared gene expression as a function of the duration of the SMG exposure. In CTL rats, 8 genes were significantly affected by the extensive duration (CTL 21 *vs* 7 days; *Celsr3, Ephb1, Lpar1, Nfib, Ulk4, Erbb3, Marcks* and *Slc1a1*; Fig. 8e and Table 2). The expression of 5 of these genes was also altered in SMG rats (Table 2; *Celsr3, Ephb1, Lpar1, Nfib, Ulk4*), indicating that the altered expression of these genes is not specific to the simulated microgravity condition but could be related to potential side effects of longer isolation in individual cages. Notably, the duration of SMG exposure specifically increased the expression of 7 genes (SMG 21 *vs* 7 days; *Btbd1, Erbb4, Grin2a, Myd88, Nfia, Prdm12, Psen1*; Fig. 8f and Table 2). However, only *Prdm12* expression was highly increased in SMG rats when the SMG period was extended to 21 days (|log2FC| > 2). Results are summarized in the Venn’s diagram (Fig. 8g).

**Table 2:** SMG and time effects on the expression of genes included in the neurogenesis GO term list revealed by RNAseq

		SMG *vs* CTL 7d		SMG *vs* CTL 21d		CTL 21d *vs* 7d		SMG 21d *vs* 7d	
	Gene name	Log2FC	*p* value	Log2FC	*p* value	Log2FC	*p* value	Log2FC	*p* value
Affected only by SMG 7d	*Ascl1*	**0.743**	**0.008**	0.081	0.76	0.318	0.26	−0.344	0.21
	*Celsr1*	**0.913**	**0.044**	0.588	0.19	0.667	0.14	0.342	0.45
	*Prdm8*	**0.382**	**0.045**	0.140	0.46	−0.063	0.74	−0.305	0.11
	*Xrcc5*	**−0.355**	**0.02**	−0.169	0.26	−0.043	0.78	0.144	0.35
Affected by both SMG 7d and 21d	*Fabp7*	**−0.474**	**0.029**	**−0.503**	**0.02**	0.261	0.23	0.232	0.28
	***Mesp1***	**−2.176**	**0.013**	**−2.884**	**0.004**	**−0.384**	**0.61**	**−1.092**	**0.32**
	*Nfix*	**0.897**	**0.016**	**0.813**	**0.03**	0.382	0.31	0.298	0.43
	*Smarca4*	**0.215**	**0.005**	**0.196**	**0.01**	−0.109	0.15	−0.128	0.09
	*Tuba1a*	**−0.354**	**0.047**	**−0.360**	**0.043**	−0.108	0.55	−0.114	0.52
Affected only by SMG 21d	*Adgrv1*	−0.165	0.25	**−0.499**	**0.0005**	0.227	0.11	−0.108	0.45
	*Ercc6*	0.159	0.34	**0.340**	**0.04**	−0.111	0.5	0.070	0.67
	*Ift20*	−0.481	0.085	**−0.677**	**0.015**	−0.235	0.4	−0.431	0.12
	*Nav1*	0.253	0.37	**0.579**	**0.039**	0.160	0.57	0.486	0.083
	*Nav2*	0.128	0.54	**0.568**	**0.006**	−0.294	0.16	0.145	0.48
	*Ndufs2*	−0.017	0.84	**−0.184**	**0.029**	0.161	0.056	−0.006	0.94
	***Pcsk1***	0.254	0.75	**1.940**	**0.011**	−0.520	0.52	1.166	0.11
	*Tacc1*	0.243	0.13	**0.322**	**0.045**	0.006	0.97	0.085	0.59
	*Trak1*	0.244	0.14	**0.461**	**0.005**	−0.167	0.31	0.050	0.76
	***Wnt1***	0.704	0.38	**2.123**	**0.01**	−0.883	0.31	0.536	0.47
Affected by time and SMG	*Prdm16*	−0.255	0.45	**0.724**	**0.029**	−0.095	0.77	**0.883**	**0.008**
Affected only by time in SMG	*Btbd1*	−0.010	0.92	0.082	0.4	0.161	0.1	**0.254**	**0.01**
	*Erbb4*	0.148	0.84	0.632	0.36	1.213	0.09	**1.696**	**0.016**
	*Grin2a*	−0.157	0.77	0.493	0.35	1.001	0.057	**1.651**	**0.002**
	*Myd88*	−0.304	0.085	0.018	0.92	0.023	0.9	**0.345**	**0.048**
	*Nfia*	−0.023	0.94	0.549	0.064	0.191	0.52	**0.762**	**0.01**
	***Prdm12***	−2.860	0.1	1.563	0.3	−0.758	0.63	**3.665**	**0.034**
	*Psen1*	0.238	0.36	0.503	0.050	0.283	0.28	**0.547**	**0.034**
Affected by time in both CTL and SMG	*Celsr3*	0.117	0.38	0.244	0.07	**−0.747**	**<0.0001**	**−0.620**	**<0.0001**
	*Ephb1*	0.064	0.66	0.029	0.84	**0.340**	**0.019**	**0.304**	**0.036**
	*Lpar1*	0.021	0.89	−0.070	0.65	**0.397**	**0.009**	**0.306**	**0.044**
	*Nfib*	0.599	0.3	0.824	0.15	**1.354**	**0.019**	**1.579**	**0.006**
	*Ulk4*	−0.004	0.98	−0.186	0.38	**−0.424**	**0.043**	**−0.606**	**0.004**
Affected only by time in CTL	*Erbb3*	0.158	0.42	0.108	0.58	**0.433**	**0.028**	0.383	0.051
	*Marcks*	−0.095	0.26	−0.153	0.068	**0.174**	**0.039**	0.115	0.17
	*Slc1a1*	−0.133	0.21	−0.057	0.59	**−0.233**	**0.026**	−0.157	0.14

Bold values indicate *p* < 0.05.

### Effects of exercise on the neurogenesis related gene expression in the hippocampus

**Fig. 9:**
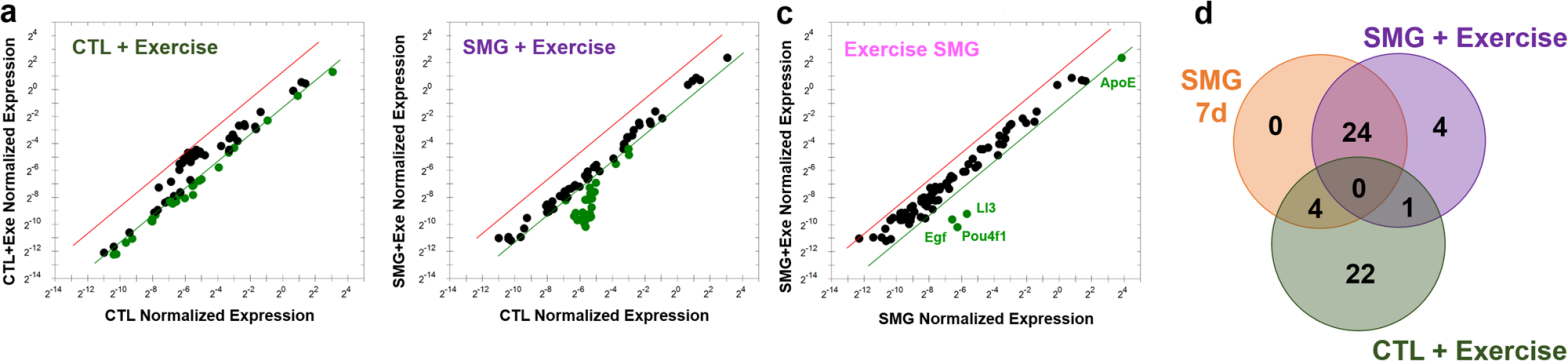
Physical exercise modulates neurogenesis related gene expression in hippocampus by RTqPCR. **a**, **b** Comparison of normalized expression of genes in CTL and in SMG conditions, respectively. Comparisons are made with control rats. **c** Comparison of normalized expression of genes of rats exposed to exercise in SMG conditions. Comparisons are made with SMG rats not exposed to exercise. Dots represent the tested genes with a fold change threshold = 2.5 (green dots on the right green line are downregulated). Graphs are generated by gene study analyses with CFX software (Bio-Rad). **d** Venn’s diagram to segregate gene populations affected by experimental conditions.

RTqPCR analyses revealed that physical exercise decreased the expression of 27 genes involved in neurogenesis in the hippocampus of CTL rats (Fig. 9a and Table 3). In SMG rats exposed to physical exercise, 39 genes showed downregulated expression (Fig. 9b and Table 3). Physical exercise induced a lower expression of *ApoE*, *Ll3*, *Pou4f1* and *Egf* in the hippocampus of SMG rats exposed to exercise than in rats exposed only to SMG (Fig. 9c). In comparison with the control condition, the Venn’s diagrams (Fig. 9d) showed that 22/27 genes were exclusively regulated by physical exercise; 24/28 genes were affected by SMG independently of exposure to the exercise countermeasure. These results suggest that exercise and SMG regulated differentially the genes implicated in neurogenesis, and that exercise was not able to reverse the SMG effect on gene expression. In addition, 4 genes (*Cdk5rap2*, *Hdac4*, *Grin1* and *Gpi*) were downregulated similarly by SMG and physical exercise; the gene *Mdk* was less expressed by physical exercise independently of SMG exposure; and 4 genes were regulated by exercise only in rats exposed to SMG (*Egf*, *Creb1*, *Pou4f1* and *Ll3*). Gene populations can be segregated by the modulation of their expression. Accordingly, physical exercise decreased the expression of *Mdk* and *ApoE* in CTL and SMG groups, exacerbated the decrease of *Cxcl1* expression, increased in CTL and decrease in SMG the expression of the genes *Egf*, *Ll3* and *Pouf4f1*, and finally had no effect or only reversed partially the effect of SMG for the others.

**Table 3:** List of genes downregulated by physical exercise in control and rats exposed to SMG during 7 days by RTqPCR analysis

CTL Exe *vs* CTL 7D		
Gene symbol	Gene ID	Gene name
*Actb*	81822	Actin
*Apbb1*	29722	Amyloid beta A4 precursor protein-binding family B n°1
*ApoE*	25728	Apolipo protein E
*App*	54226	Amyloid β A4 protein
*Cdk5rap2*	286919	Cdk5 regulatory subunit-associated protein 2
*Drd2*	2520	Dopamine receptor D2
*Fgf2*	54250	Fibroblast growth factor 2
*Flna*	293860	Filamin-A
*Gpi*	292804	Glucose-6-phosphate isomerase
*Grin1*	24408	Glutamate NMDA receptor subunit ζ-1
*Hdac4*	363287	Histone deacetylase 4
*Hsp90ab1*	301252	Heat shock protein HSP90-β
*Mdk*	81517	Midkine
*Mef2c*	499497	Myocyte enhancer factor 2C
*Mll1*	315606	Myeloid/lymphoid or mixed-lineage leukemia 1
*Nf1*	24592	Neurofibromin
*Notch1*	25496	Neurogenic locus notch homolog protein 1 precursor
*Notch2*	29492	Neurogenic locus notch homolog protein 2 precursor
*Nrp1*	246331	Neuropilin-1
*Nrp2*	81527	Neuropilin-2
*Pafah1b1*	83572	Platelet-activating factor acetylhydrolase IB subunit α
*S100b*	25742	Protein S100-B
*Slit2*	360272	Slit homolog 2 protein precursor
*Stat3*	25125	Signal transduser and activator of transcription 3
*Tbp*	117526	Taba-box-binding protein
*Th*	25085	Tyrosin 3 monooxygenase
*Tnr*	3886	Tenascin R

SMG Exe *vs* CTL 7D		
Gene symbol	Gene ID	Gene name
*Adora1*	29290	Adenosine receptor A1
*Adora2a*	25369	Adenosine receptor A2a
*Ascl1*	64186	Ashaete-scute homolog 1
*Bcl2*	24224	Apoptosis regulator BCl2
*Bmp8a*	680931	Bone morphogenetic protein 8a precursor
*Cdk5r1*	116671	Cyclin dependent kinase 5 activator 1
*Chat*	290567	Choline O-acetyl transferase
*Creb1*	81646	Cyclic AMP-responsive element-binding protein 1
*Cxcl1*	81503	Growth-regulated α protein precursor
*Dll1*	84010	Delta like protein 1 precursor
*Efnb1*	25186	Ephrin-B1 precursor
*Egf*	25313	Pro-epidemal growth factor precursor
*Ep300*	620036	E1A binding protein p300
*Erbb2*	24337	Receptor tyrosin-protein kinase erbB-2 precursor
*Gdnf*	25453	Glial cell line-derived neurotrophic factor precursor
*Hey1*	155437	Hary/enhancer-off-split related with Yrpw motif protein 1
*Hey2*	155430	Hary/enhancer-off-split related with Yrpw motif protein 2
*Il3*	24495	Interleukin 3 precursor
*Lif*	60584	Leukemia inhibitory factor precursor
*Mdk*	81517	Midkine
*Neurog1*	29410	Neurogenin-1
*Neurog2*	295475	Neurogenin-2
*Nog*	25495	Noggin precursor
*Pax3*	114502	Paired box 3
*Pax6*	25509	Paired box protein 6
*Pou3f3*	192109	Pou domain, class 3, transcription factor 3
*Pou4f1*	114503	Pou domain, class 4, transcription factor 1
*Shh*	29499	Sonic hedgehog protein precursor
*Sox2*	499593	Sry-box 2
*Sox8*	302993	Transcription factor Sox8

**Fig. 10:**
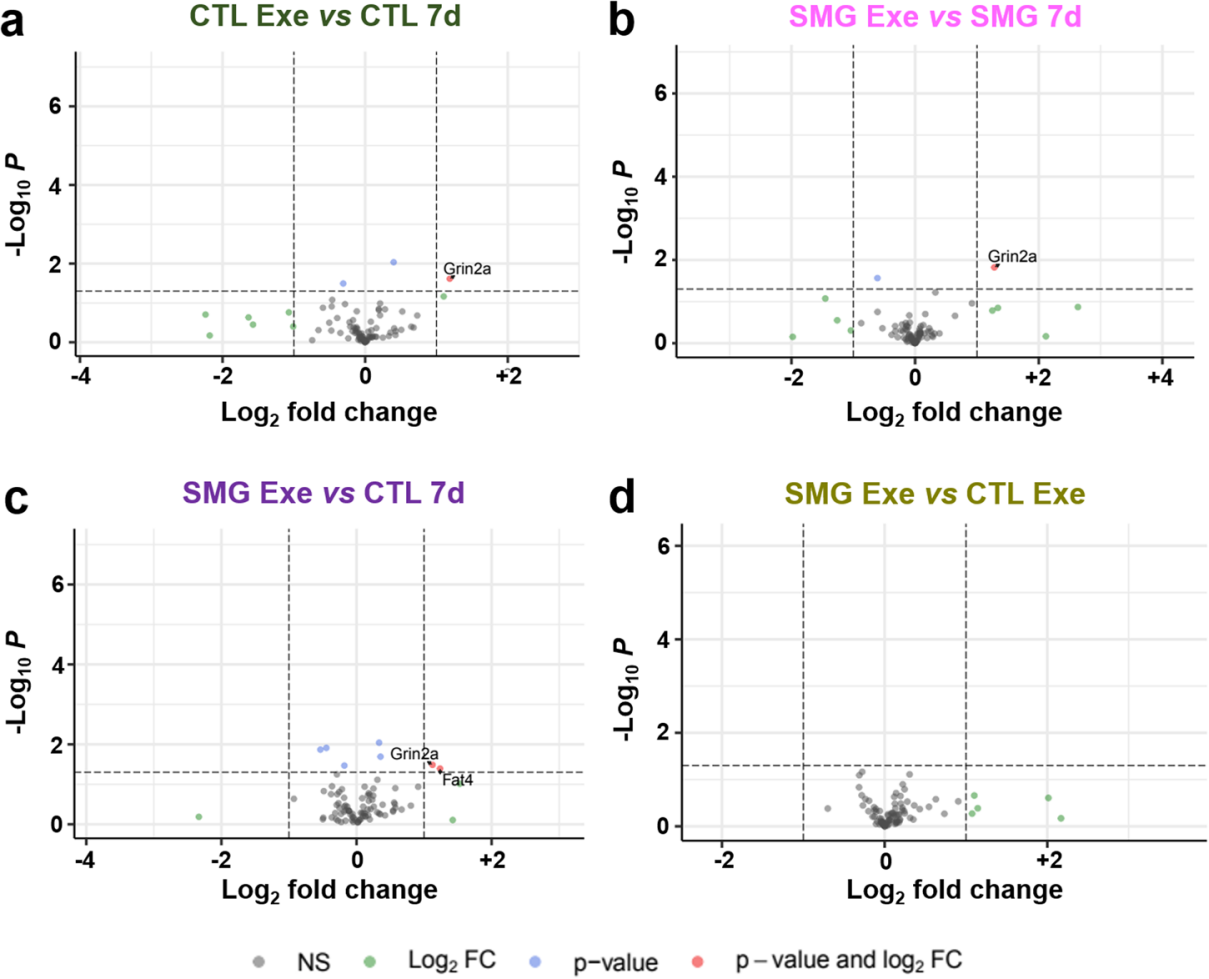
Physical exercise modulates neurogenesis related gene expression in hippocampus by RNAseq. Volcano plots of expression of genes of rats exposed to exercise in CTL (**a**) or SMG (**b**) conditions. Volcano plot of expression of neurogenesis related genes in hippocampus of rats exposed to exercise in SMG conditions compared with CTL rats without (**c**) or with (**d**) exercise. In gray (NS) is the group of the non-affected genes, in blue (*p* < 0.05) the expression of gene is significantly affected, in green the fold is change more than twice (|LnFC| > 2) and finally in red are indicted the gene expression significantly and highly changed (*p* < 0.05 and |LnFC| > 2).

RNAseq analyses revealed that the expression of *Adgrv1* was significantly decreased by physical exercise only in CTL rats (Table 3). The expression of *Dagla* and *Grin2a* was increased by physical exercise in both SMG and CTL rats but only *Grin2a* expression was also increased by exercise in comparison with SMG rats not exposed to exercise (Fig. 10c and Table 3).

While 7 days of SMG exposure decreased the expression of 4 genes involved in neurogenesis (Fig. 8c and Tables 2–4; *Mesp1, Xrcc5*, *Tuba1a* and *Fabp7*) and increased the expression of 5 other genes (Fig. 8c and Tables 2–4; *Ascl1, Celsr1, Prdm8, Nfix* and *Smarca4*), the expression of *Tuba1a* and *Fabp7* was similarly decreased in SMG rats performing exercise (Tables 3 and 4). While the expression of Ascl1 was decreased in the SMG condition, physical exercise reversed its expression (Tables 3 and 4). Finally, the expression of *Brinp1*, *Marcks* and *Fat4* was changed in SMG rats submitted to exercise compared to the CTL condition.

**Table 4:** Exercise and SMG effects on the expression of genes included in the neurogenesis GO term list revealed by RNAseq

		SMG *vs* CTL 7d		CTL + Exe *vs* CTL 7d		SMG + Exe *vs* CTL 7d		SMG + Exe *vs* SMG 7d		SMG + Exe *vs* CTL + Exe	
	Gene name	Log2FC	*p* value	Log2FC	*p* value	Log2FC	*p* value	Log2FC	*p* value	Log2FC	*p* value
Affected by exercise in CTL	*Adgrv1*	−0.165	0.25	**−0.308**	**0.032**	−0.215	0.13	−0.050	0.73	0.093	0.52
Affected by exercise in both	*Dagla*	0.297	0.052	**0.399**	**0.009**	**0.355**	**0.02**	0.058	0.71	−0.044	0.78
	*Grin2a*	−0.157	0.77	**1.186**	**0.024**	**1.125**	**0.032**	**1.282**	**0.015**	−0.061	0.91
Affected only by SMG without exercise	*Celsr1*	**0.913**	**0.044**	0.158	0.73	0.305	0.5	−0.608	0.18	0.147	0.75
	*Mesp1*	**−2.176**	**0.013**	−1.071	0.17	−0.927	0.23	1.249	0.16	0.144	0.86
	*Nfix*	**0.897**	**0.017**	0.520	0.17	0.575	0.13	−0.323	0.39	0.054	0.88
	*Prdm8*	**0.382**	**0.045**	0.277	0.15	0.248	0.19	−0.134	0.48	−0.030	0.88
	*Smarca4*	**0.215**	**0.005**	0.079	0.3	0.120	0.12	−0.095	0.21	0.041	0.59
	*Xrcc5*	**−0.355**	**0.02**	−0.013	0.93	−0.290	0.056	0.066	0.67	−0.276	0.068
Affected by SMG and exercise in SMG condition	*Tuba1a*	**−0.354**	**0.047**	−0.135	0.45	**−0.447**	**0.012**	−0.092	0.61	−0.312	0.08
	*Fabp7*	**−0.474**	**0.029**	−0.220	0.3096	**−0.535**	**0.013**	−0.060	0.78	−0.315	0.15
	*Ascl1*	**0.743**	**0.008**	−0.084	0.77	0.133	0.64	**−0.610**	**0.027**	0.217	0.45
Affected by exercise in SMG	*Brinp1*	0.170	0.19	0.209	0.1	**0.335**	**0.009**	0.164	0.19	0.125	0.33
	*Marcks*	−0.095	0.26	−0.118	0.16	**−0.180**	**0.034**	−0.085	0.32	−0.061	0.47
	*Fat4*	1.041	0.085	1.101	0.068	**1.237**	**0.04**	0.195	0.74	0.136	0.82

Bold values indicate *p* < 0.05.

## Discussion

The present study found significant reduction of newborn cell proliferation in DG and newborn cell survival in DG and SVZ after 7 days of exposure to SMG in male rats. This neurogenesis impairment was only transient as it was no longer observable after a SMG period of 21 days. Physical exercise used as a countermeasure was able to restore adult neurogenesis in the DG of rats exposed to SMG.

It is well-known that microgravity impacts the soleus muscle in astronauts during spaceflights and in animal models of SMG^42^. Reduction in muscle mass observed in the present study is in accordance with several previous investigations that have found muscle atrophy after 7 days^45–47^, 2 weeks^37,48–50^ or one month of hindlimb suspension^51^. Physical exercise administered for two weeks in an activity wheel can counteract this deleterious effect^37^. Concurring with this, we also observed an increase in the soleus muscle weight of our rats exercising for 3-week on a running treadmill. We did not observe any difference in the distance covered by control and SMG rats during our imposed treadmill sessions. However, in spontaneous locomotor activity tests during which animals exposed to SMG can freely access an activity wheel, detailed analyses of locomotion patterns showed multiple long-term deficits, such as a shorter distance covered, a lower number of movements and their duration, and a reduced vertical activity^37^. These alterations were observed immediately upon the end of SMG exposure and persisted 2 weeks later, indicating that the effects of SMG on locomotion are long-lasting^37^.

Simulated microgravity in the HU model leads to a reduction in weight gain of rats. This alteration in body weight during suspension has been documented on several previous occasions in rodents^24,37,45,46,48,49,51–54^. As previously shown^48,52,54^, the consumption of water and food in our study was not impacted by simulated microgravity during hindlimb suspension. It is interesting to note that, despite identical food and water consumption, rodents subjected to SMG show lower weight gain at least at the start of the procedure. This suggests either metabolic changes^55^, or an increase in energy expenditure^49,56^ in rats undergoing SMG. In our experiments, EdU injections led to a transient reduction in food and water consumption, associated with reduced weight gain in both CTL and SMG rats. Since these injections represent a significant intake of body fluid, it may explain the drop in water consumption the day after the injections. In addition, injections could generate a stress, known to induce hypophagia^57^.

We observed that SMG impaired newborn cell proliferation in the DG by 40% following a 7-day exposure period to HU but not after a shorter period of 6 or 24 h. It has been previously observed that SMG decreased by 50% the newborn cell proliferation in DG after 2 weeks exposure in the HU model in rats^37^, by two third after 2 weeks exposure in the HU model in mice^36^ or by 40% after 6 weeks exposure in the head-down bed rest model in rhesus macaque^58^. On the contrary, 3-day exposure to SMG using the HU model did not decrease the number of Ki67^+^ cells in the DG^59^. These results indicate that it does require some time of exposure to microgravity before effects on the proliferation of new cells can be observed. Nevertheless, in our study, no effect was observed on newborn cell proliferation after 21 days of exposure to SMG. In addition, we observed that the levels of newborn cell proliferation were not similar in CTL rats between 7- and 21-days of SMG exposure (although the difference (*p* < 0.12) failed to reach significance). This lack of effect after 21 days of exposure to SMG could be masked by the reduced number of newborn cells produced in CTL rats. This reduction in newborn cell proliferation in CTL rats may be due to social isolation, which can alter the proliferation of the newborn cells in the hippocampus^33,34^.

We also observed that SMG reduced newborn cell survival in DG by a third and in SVZ by 20% after 7-day exposure to HU. However, no effect of SMG on adult neurogenesis was observed after 21 days of exposure to HU, indicating a transient effect. Exposure to SMG in HU model during 3 days induced a decrease in number of immature newborn neurons in the DG in rats^59^. In rhesus macaques, it has been observed a 50% decrease of newborn cell survival in the DG after 6 weeks exposure to SMG in the head-down bed rest model^58^. However, in this last study, monkeys were severely restrained on the bed, which can induce stress associated with increase in plasma hormones involved in response to stress, known to reduce adult hippocampal neurogenesis^60^. Moreover, in humans, the plasma level of cortisol was increased in volunteers during bed rest experiments^61^. In our HU model, any major stress effects on adult neurogenesis appear unlikely because we did not observe an increase of the corticosterone level in rats submitted to SMG. In addition, the effect of SMG on newborn cell survival observed in HU rats was not due to social isolation, as no difference was observed between isolated and group-housed rats (Supplementary Fig. 3). Finally, no effect of SMG was observed in the OB. This is consistent with previous study showing no difference on the density of newborn neurons in OB of female mice after a 13-day spaceflight^62^.

Adult neurogenesis is known to play a number of roles in the brain, particularly in cognitive functions. Adult olfactory neurogenesis is implicated in social behavior linked to innate responses to odors^63^, in odor detection, discrimination and spontaneous recognition^64^, or in olfactory learning and memory^65^. Adult hippocampal neurogenesis is largely involved in learning and memory processes^25^, including spatial memory^66–69^ and in pattern separation function of the DG^70,71^. In our study, we showed that adult neurogenesis is strongly but transiently impaired, mainly in the hippocampus. This could lead to more or less significant memory deficits, which would be interesting to quantify precisely in different tasks known to involve adult neurogenesis and which require considerable cognitive processing.

The effect of SMG on adult neurogenesis seems to be attributable to modifications in biological and molecular properties of neural stem cells. This cell type derived from the SVZ of mice exposed to HU model during 2 weeks showed reduced proliferation capability, altered cell cycle and incomplete differentiation/maturation^36^. However, 3-day exposure to SMG in the HU model is not enough to induce deregulation of the expression of *Sox2*^59^ which controls proper self-renewal of neural stem cells^72^. In addition, HU-induced SMG leads to a decrease in the cerebral expression of several factors known to play a role in adult neurogenesis regulation such as NR2A/NR2B NMDA receptors^19,20,59^, insulin-like growth factor-1 (IGF-1)^73^, brain derived neurotrophic factor (BDNF)^15,20,24^ or p-CREB (cAMP-response element binding protein)^15,19^.

The results of our RTqPCR and transcriptomic analyses suggested that the effects of the exposure to 7 and 21 days to hindlimb suspension affected differently the neurogenesis related gene expression. After 7 days of SMG, some genes implicated in neurogenesis were partially downregulated, suggesting a global impairment of neurogenesis pathways. But after 21 days of SMG, the expression of these genes was upregulated. This result suggests that during the first week of SMG, the adult neurogenesis process is globally impacted by simulated microgravity, but after this initial period, some compensatory mechanisms were engaged to restore the original level of hippocampal integration of newborn neurons. In comparison, after 28 days of hindlimb suspension, proteomic analysis suggested that the expression of few (close to 10%) proteins involved in neurogenesis were affected^74^.

In our analysis, we observed that simulated microgravity regulated several genes known to be involved in neurogenesis (Table 4). It has been shown that these genes, as *EdbB4, Ascl1, Pax6, EphB, Cxcl1, Bcl2, Tuba1a or Nfix*, are notably involved in the organization of the neurogenic niche^75^, in neural stem cells quiescence^76,77^ and maintenance^78–80^, in neural stem cell self-renewal^81,82^, in progenitor cell proliferation^82–87^ and differentiation^78^, in neuroblast maturation and survival^88,89^, in neurite growth^90^, in neuronal polarization^91^, in neuronal migration^85,92–94^, or in the integration of newly generated neurons^78,95^.

To better understand the regulation of gene expression induced by microgravity, the experiments should be investigated more precisely by single cell RNAseq^96,97^ and localization experiments based on the result of the bulk analysis reported here. In fact, SMG should have different effects on neuronal lineages depending also on the duration of SMG exposure. Moreover, many other pathways concerning neuron activity should be analyzed in more details, including the role of inflammatory factors, microglia and oligodendrocytes and factors released by endothelium^98,99^.

Physical activity is the most effective way to limit the adverse effects of microgravity on the human body during spaceflight^38,39^, including treadmill training (which provides cardiovascular, muscular and skeletal exercise)^100^, and represent an essential part of the astronauts’ daily routine. Physical exercise (wheel or treadmill) is a well-proven neurogenic stimulus in rodents, promoting neuronal progenitor proliferation and newborn cell survival, and influencing the morpho-functional maturation process of newborn neurons^37,101–110^.

Treadmill speed has been shown to induce different effects on adult hippocampal neurogenesis. At low- or mild-intensity speed, i.e., between 10 and 30 cm/s, several studies have shown a 1.5 to 2-fold increase in the level of newborn cells produced and surviving in the DG^101,108–110^. However, intense-intensity speed, i.e., superior to 40 cm/s, induces an increase of the proliferation of newborn cells but no effect on their long-term survival^102,108^. Based on these previous results and on the fact that rats would be subjected to exercise during SMG exposure, we chose a mild-intensity speed for our study. In control rats, we reproduced the 1.5 to 2-fold increase of the newborn cells’ survival in the DG (Fig. 6), indicating that our exercise protocol has the expected effect on adult neurogenesis. Moreover, we observed a 1.5-fold increase of the number of newborn neurons in the OB. This result is surprising as exercise has been shown to induce an increase in adult neurogenesis in the hippocampus but not in the OB^111^. In our study, the animals were isolated, leading to a reduction in social interactions and a depletion of olfactory stimuli. During treadmill sessions, the animals may have left a variety of olfactory marks in a confined space possibly leading to an increase in olfactory stimulation and consequently an increase in adult neurogenesis in the OB. This effect has already been observed in a confined space module left on the ground^62^.

The major finding of our study is that physical exercise completely restores newborn cell survival impaired by SMG in the DG and SVZ. A positive effect of physical exercise has already been observed in rats exposed to the HU model, without however completely restoring newborn cell proliferation level in DG or SVZ^37^. The difference observed between this previous study and ours is probably due to the type of exercise, i.e., activity wheel *vs* treadmill, its intensity, i.e., free access *vs* imposed speed, and duration, i.e., 3 h/day during 2 weeks *vs* 30 min/day during 3 weeks. Moreover, in our experiment, rats underwent exercise sessions before and during SMG exposure, whereas in the study by Yasuhara and colleagues, rats were exposed to physical exercise only after SMG exposure^37^. In addition, they did not observe a positive effect on adult neurogenesis when SMG exposure was discontinued for 2 weeks without physical exercise^37^. This indicates that adult neurogenesis remains impacted for a long time after exposure to SMG. Thus, it is therefore unlikely that in our protocol, the observed effect of physical exercise on adult neurogenesis survival in a SMG context is linked to the fact that the animals are no longer suspended for 30 min a day for exercise session. Moreover, our results also confirm those obtained with dynamic foot stimulation of the plantar surface in rats during SMG exposure that also prevents the reduction of DCX cells in the DG^59^. Altogether, it seems that preventive exposure to physical exercise and/or during SMG exposure are more effective in preventing the impact of SMG on adult neurogenesis than during the recovery phase after SMG exposure.

Molecular mechanisms underlying the effect of physical exercise on adult neurogenesis are related to many factors, such as BDNF^103,112^, NMDA receptors^112^, fibroblast growth factor 2 (FGF-2)^113^, vascular endothelial growth factor (VEGF)^114^ or IGF-1^109,115^. The neurogenic niche in the DG is organized in close association to endothelial cells forming a supportive vascular niche in which VEGF and IGF-1 serve as supporting factors^109,114,115^. Physical exercise in rats submitted to simulated microgravity in the HU model maintains levels expression of NMDA receptors and VEGF comparable to control rats^37,59^. In this study, the expression of these genes was not affected by SMG. The transcriptomic analysis indicated that the effects of the exercise did not reverse strictly the effects of SMG on neurogenesis related genes but may have acted *via* modulation of different transduction pathways.

There are some limitations to this study but it proposes several perspectives for future research. This study primarily focused on the effects of SMG on the proliferation and survival of newborn neurons in the adult brain of resting rats. How SMG may affect adult neurogenesis induced by behavioral challenges deserves further investigations. As previous discuss, it is well known that adult neurogenesis plays an important role in learning and memory processes^25^, including the formation of enduring memories^66–68^ or the maintenance and update of remote memory reconsolidation^116^. For instance, it will be particularly interesting to explore how SMG may affect the functional integration of new neurons in relation to learning and memory functions in rats exposed to HU and submitted to hippocampal-dependent tasks.

In this study, we limited our transcriptome analysis to adult neurogenesis. It would be relevant to extend the analysis to the entire hippocampal transcriptome and to focus more specifically on the genes involved in learning and memory functions. iTRAQ-based proteomics analyses identified changes in metabotropic and ionotropic glutamate receptor pathway associated with impairment in spatial learning and memory in rats exposed to 28 days of SMG^13^. Also, after 7 days of hindlimb suspension in mice, proteomic analysis in hippocampus revealed major losses of proteins involved in metabolism or in structural proteins^117^.

To better adapt future physical exercise protocols during spaceflights and long-term missions, the effects of exercise as a countermeasure should be more thoroughly characterized. In particular, it would be interesting to determine whether physical exercise has a differential impact on adult neurogenesis before or during exposure to SMG. Moreover, the effects of spaceflights on physiological parameters depend on the duration of exposure to microgravity. To restore physiological functions altered by spaceflights, the optimal exercise modalities in terms of intensity and duration before and/or during spaceflight, and including individual parameters concerning muscular capacities, should be identified to design the most appropriate exercise protocols.

In addition, it will be important to investigate additional contributing factors likely to alter adult neurogenesis during spaceflights. Combining several stress factors such as radiations^118^ or hypomagnetic field^119^ will be needed to better model the environmental conditions experienced by astronauts during space missions. Another major point to consider is the sex effect. Indeed, adult neurogenesis is not regulated in the same manner in male and female rodents and the effects of spaceflights are not exactly similar. RNA-Seq analysis could be a pertinent tool to investigate the cross effects between sex and SMG on adult neurogenesis.

## Materials and methods

### Animals

Adult male Long Evans rats (Charles River, 7 weeks on arrival, *n* = 124) were use throughout. Rats were initially housed in groups, 2 per cage, according to the calculation of the size of the cage as a function of their weight (model GR900, surface: 904 cm², Techniplast) and were maintained under standard condition in a temperature- and humidity-controlled experimental room. The room was under a 12 h light/dark cycle (light onset 7.30AM) and experiments were performed during the light phase of the cycle. Food (A04 product, SAFE) and tap water were available *ad libitum* during the study. The rats were handled once daily to minimize stress and to familiarize them with the male and female experimenters. Enrichment was also used to reduce stress (piece of wood to chew on, ref 14205, cardboard tunnel ref 14179 from PLEXX and wood chips ref SAFE 3-4S from SAFE). All experiments were in accordance with the European Communities Council Directive (2010/63/EU Council Directive Decree) regarding the care and use of laboratory animals. The procedures were efforts were made to minimize the number of animals and their suffering, and to maximize their well-being during the experiments. The experimental design on animal was approved by ethical committee CE50 (Bordeaux, France) and French ministry of Agriculture (2020011015121788).

### Experimental model for simulated microgravity

To simulate microgravity, we used the HU rat model (Imetronic®, Fig. 1a), modified from the Morey-Holton method^8,10,120^ and described in our previous study^16^. Briefly, the tails of the rats were cleaned and protected with medical adhesive tape. A metal ring with a flexible cord was attached to the rat’s tails with tape. The cord was clipped on a pulley attached to a mobile metal bar inserted in the roof of the cages. Rats were able to move freely in a 360° arc in the cage (surface 1050 cm²) with their forelimbs. The hindlimbs were kept above the cage floor. The head-down tilt position was near 40–45°.

### Experimental design

After acclimation to the vivarium conditions (PIVE-EXPE, University of Bordeaux, agreement A32-063-940) and daily handling for 10 days, the rats were divided into several groups to evaluate adult neurogenesis (*n* = 97) and gene expression (*n* = 24). After an acclimation period in individual cages for 3 days, the hindlimb-suspended rats were maintained under simulated microgravity for 6 h to 21 days. For the adult neurogenesis study, rats were exposed to SMG for 6 h (CTL *n* = 8, SMG *n* = 8), 24 h (CTL *n* = 8, SMG *n* = 8), 7 days (HC *n* = 10, CTL *n* = 8, SMG *n* = 8), or 21 days (HC *n* = 6, CTL *n* = 6, SMG *n* = 7) after nucleoside analog (EdU or BrdU) injections. Physical exercise was used as a countermeasure in rats exposed 7 days to SMG (CTL Exe *n* = 10, SMG Exe *n* = 10). To limit the number of rats used in our study (3R rule), some rats were injected with both EdU and BrdU (Fig. 2a), so two cohorts of newborn cells could be tracked in the same animals. In this way, we can study both survival and proliferation of newborn cells within the same population of animals. Moreover, the injection of EdU and BrdU in same animals is the best solution to possibly correlated effects on the beginning and end of the suspension period. For the gene expression study, rats were exposed to SMG for 7d (CTL *n* = 4, SMG *n* = 4, CTL Exe *n* = 4, SMG Exe *n* = 4) or 21d (CTL *n* = 4, SMG *n* = 4). The animals were monitored daily to ensure access to water and food and verify their well-being and that their hindlimbs were not touching the floor grid. The control rats were kept isolated in standard cages with an identical grid to suspended cages to ensure similar sensory inputs between conditions. We did not observe any suffering in the hindlimb-suspended rats. The suspension was stopped for 2 out to 49 rats due to tail swelling during the experiment after 5 and 13 days of hindlimb suspension. Moreover, one rat died before the beginning of the experiment. These 3 rats were excluded from the study.

### Physical exercise (treadmill)

Prior to the experiment, rats (*n* = 28 – 20 rats for the adult neurogenesis study and 8 rats for the gene expression study) were habituated to the treadmill (Imetronic®, Fig. 5a) for 2 days during which they were allowed to explore the treadmill at low speeds (5–10 cm/s) for 10 min. The speed of the treadmill was then increased to 20 cm/s for three consecutive daily sessions of 10 min. Rats in the exercise groups were placed in a treadmill and underwent forced running for 30 min a day for 3 weeks: 2 weeks prior to the SMG protocol and 1 week during the SMG exposure. During the first week, the speed was set at 25 cm/s. During the second and third weeks, the speed was increased to 30 cm/s. Rats were carefully monitored and the speed of the treadmill was adapted to accommodate the behavior of the animals during the running session, in particular for the rats under SMG, to avoid stress and injuries and to maintain the animal welfare. The speed of the treadmill and/or the duration of the sessions was adapted for 12 rats (4 CTL and 8 SMG – minimum 15 cm/s – minimum 15 min/session), i.e., 42.9% of the rats involved in the physical exercise protocol, for 1 to 6 running sessions. Running experiments take place during the light phase between 8.30 AM and 5.00 PM. Control rats remained in their home cage near the treadmill.

### Physiological measurements

The weight and body temperature of the rats and their food/water consumption was recorded daily. For rats submitted to physical exercise in the treadmill, measures were taken after the exercise session. Glycemia was measured in blood with the Accu-check Performa device (Roche), the day of the isolation, just before HU suspension and just after the end of the suspension procedure 6 h, 24 h, 7 days or 21 days later. Blood was collected by puncturing the end of the tail of each animal. The corticosterone concentrations were measured by ELISA (K014-Arbor Assay LLC) following the instructions of the supplier, in the supernatant of the blood collected just before perfusion and after centrifugation at 10,000 rpm for 10 min at 4 °C. The supernatant was stored at −20 °C until use. Results were read using a microplate reader (FLUOstar® Omega, BMG Labtech, Champigny-sur-Marne, France) at 450 nm. To check the effect of SMG on muscle weight, two different muscles were collected, the soleus muscle from the hind limbs and the extensor carpi radialis longus muscle from the fore limbs, in CTL (*n* = 4) and SMG (*n* = 4) rats. Muscles were weighed directly after collection on the same precision scale.

### Adult neurogenesis study

#### EdU and BrdU administration

To study adult newborn cell proliferation, rats received one intraperitoneal injection of 5-Ethynyl-2’-deoxyuridine (EdU, 200 mg/kg in 0.9% NaCl, BOC Sciences 61135-33-9) and were killed 6 (CTL *n* = 8, SMG *n* = 8) or 24 h (CTL *n* = 8, SMG *n* = 8) later. For adult newborn cell survival, rats were given two EdU injections (200 mg/kg in 0.9% NaCl) at 6 h interval on a single day and were killed 7 (HC *n* = 10, CTL *n* = 8, SMG *n* = 8, Exo CTL *n* = 10, Exo SMG *n* = 10) or 21 (CTL *n* = 4, SMG *n* = 4) days later. These rats also received one intraperitoneal injection of 5-Bromo-2’-deoxyuridine (BrdU, 200 mg/kg in 0.9% NaCl, BOC Sciences 59-14-3) 24 h before sacrifice to study adult newborn cell proliferation after several days of simulated microgravity exposure and to reduce the number of animals used for this study.

### Tissue collection and sectioning

Rats were deeply anesthetized with an intraperitoneal injection of mixed Exagon (40 mg/kg) and Lurocaïne (20 mg/kg) solution and then perfused transcardially with a 0.9% NaCl solution during 20 min and then a 4% ice-cold paraformaldehyde solution in 0.1 M phosphate buffer (PB), pH 7.4 during 30 min at 13 ml/min. Brains were removed and post-fixed overnight in the same perfusion solution at 4 °C, immersed for 5 days in PB containing 30% sucrose, frozen in chilled 2-methylbutane at −50 °C and preserved at −20 °C. Coronal serial 40-µm-thick free-floating sections were cut with a cryostat (Leica CM3050S) for the olfactory bulb (OB), SVZ and hippocampus area. Brain sections were stored at −20 °C in a cryoprotectant solution until use.

### Immunochemistry

Revelations of EdU+, BrdU+, Sox2+, GFAP+, DCX+ and NeuN+ cells were performed on floating sections. After several rinses in phosphate buffer solution (PBS 0.1 M) to remove the cryoprotection solution, sections were pre-incubated in Target Retrieval Solution (S1700, Dako) for 20 min at 80 °C. After cooling for 10 min, sections were treated with 0.5% Triton in PBS for 20 min. EdU revelation was performed using the Click-it^TM^ EdU Alexa Fluor^TM^ 555 imaging kit (C10338, Invitrogen), incubated for 30 min. For BrdU revelation, two supplementary steps were applied: sections were incubated for 15 min in 2 N HCl at room temperature to denature the DNA strands and allow the antigenic sites to become accessible to the antibody, followed by 15 min in 0.1 M Boric acid, pH 8.5 to reduced pH to a neutral value. After PBS washes, sections were incubated for 60 min in 10% goat serum (CAECHV00-0U, EuroBio), 0.2% Triton (Triton X-100, 9002-93-1, Sigma), and 0.1% BSA (04-100-812-C, Euromedex) to block nonspecific binding, and incubated overnight at room temperature in a rabbit anti-BrdU primary antibody (1:250, GTX128091, GeneTex), and/or a chicken anti-GFAP (1:4000, Ab4674, Abcam), a rabbit anti-Sox2 (1:250, GTX101507, GeneTex), a guinea-pig anti-DCX (1:2000 for OB, 1:500 for SVZ and hippocampus, Abcam ab2253), a rabbit anti-NeuN (1:2000, GTX132974, GeneTex). Sections were then incubated for 2 h at room temperature in a goat anti-rabbit secondary Cy3 antibody (1:250, A120-201C3, Bethyl), and/or goat anti-chicken Cy5 (1:500, A30-206C5, Bethyl), goat anti-rabbit 488 (1:500, A11008, Invitrogen-ThermoFisher), a goat anti-guinea-pig 488 (1:500 for OB, 1:250 for SVZ and hippocampus, A11073, Invitrogen-ThermoFisher), a goat anti-rabbit Cy5 (1:500, A120-201C5, Bethyl). Sections were rinsed, incubated 10 min with Hoechst® (1:2000) in PBS, rinsed in PB, mounted and coverslipped in Fluoromount (FP-48331, Interchim).

#### Image acquisition, quantification and analysis

All cell counts were conducted by an experimenter blind to the experimental conditions.

##### EdU+ and BrdU+ nuclei quantification

Labeled profiles in hippocampus were counted with a Leica microscope (DM6000 B) coupled with a JAI camera (Apex Series AT-140GE) and a mapping software (Mercator Pro, Explora Nova). The surface area of the DG was traced at objectives 2.5X (NA 0.07) and 10X (HC PL AP0 NA 0.4) and reported in Table 5. Counting of EdU+ and BrdU+ cells was done in the DG of the dorsal hippocampus, from Bregma −2.52 mm to −4.56 mm (The rat brain, 5^th^ edition, Paxinos & Watson), using a mean of 7 sections per animal spaced by 240 µm, at the objective 20X (PL Ap0 NA 0.6) and 40X (PL AP0 NA 0.75). The density of EdU+ of BrdU+ cells per mm² in the DG was obtained by dividing the number of EdU+ or BrdU+ cells detected by the surface counted.

**Table 5:** Surface (mm²) analyzed in DG, SVZ and OB for the study of EdU+ and BrdU+ nuclei quantification

Adult neurogenesis			Structure			
			DG	SVZ	OB	
Proliferation	EdU 6 h	CTL	0.29 ± 0.01	0.15 ± 0.01		Supplementary Fig. 2B, C
		SMG	0.29 ± 0.01	0.15 ± 0.01		
	EdU 24 h	CTL	0.35 ± 0.02	0.15 ± 0.01		Fig. 3B, C & Supplementary Fig. 2B, C
		SMG	0.29 ± 0.02	0.15 ± 0.01		
	BrdU 7d	CTL	0.26 ± 0.01	0.12 ± 0.02		Fig. 3E, F
		SMG	0.26 ± 0.01	0.09 ± 0.002		
	BrdU 21d	CTL	0.25 ± 0.01	0.10 ± 0.01		
		SMG	0.24 ± 0.01	0.10 ± 0.01		
Survival	EdU 7d	HC	0.36 ± 0.02	0.15 ± 0.01	7.05 ± 0.17	Figs. 4B, D, F and 6B, D, F & Supplementary Fig. 3
		CTL	0.33 ± 0.01	0.15 ± 0.01	7.39 ± 0.19	
		SMG	0.32 ± 0.02	0.15 ± 0.01	7.46 ± 0.22	
		CTL Exe	0.35 ± 0.01	0.17 ± 0.004	7.37 ± 0.25	
		SMG Exe	0.32 ± 0.01	0.17 ± 0.02	7.46 ± 0.13	
	EdU 21d	HC	0.32 ± 0.01	0.15 ± 0.01	6.88 ± 0.34	Fig. 4B, D, F & Supplementary Fig. 3
		CTL	0.30 ± 0.02	0.18 ± 0.01	7.54 ± 0.24	
		SMG	0.30 ± 0.01	0.18 ± 0.01	7.39 ± 0.26	

Labeled profiles in OB and SVZ were counted with a slide scanner (Nanozoomer 2.0 HT, Hamamatsu) at objective 20X. The surface area of the SVZ and OB was traced using the mapping QuPath software and reported in Table 5. EdU+ and BrdU+ nuclei were automatically counted with QuPath (v0.2.3, 2020 QuPath developers, University of Edinburgh) in the OB using a mean of 10 sections spaced by 240 µm from Bregma +9 mm to +6 mm (The rat brain, 5^th^ edition, Paxinos & Watson) and in the SVZ using a mean of 9 sections per animal spaced by 240 µm from Bregma +2.28 mm to –0.6 mm (The rat brain, 5^th^ edition, Paxinos & Watson). The TRITC fluorescence was automatically detected based on a fixed threshold apply for all the animal to avoid bias. For SVZ, the fluorescence was strong and clustered in large areas of several EdU+ or BrdU+ cells. Based on this observation, the detected fluorescence surface was divided by the area of an SVZ cell (41 µm²) to obtain the number of EdU+ or BrdU+ cells. Then the number of EdU+ or BrdU+ cells was divided by the SVZ surface counted to obtain the density of EdU+ or BrdU+ cells per mm².

##### GFAP+, Sox2+, DCX+ and NeuN+ cells quantification

Sections were analyzed using a spinning disk microscope (Leica DMI8, Leica Microsystems, Wetzlar, Germany) equipped with a confocal Scanner Unit CSU-W1 T2 (Yokogawa Electric Corporation, Tokyo, Japan). Images were acquired using an 40X (HCX PL Ap0 NA 1.25) objective and a sCMOS Prime 95B camera (Photometrics, Tuscon, USA). The LASER diodes used were at 405 nm (100 mW), 488 nm (400 mW), 561 nm (400 mW) and 642 nm (100 mW). Z-stacks were done with a galvanometric stage (Leica Microsystems, Wetzlar, Germany). The system was controlled by MetaMorph software (Molecular Devices, Sunnyvale, USA). The percentage of EdU+ cells expressing GFAP+, Sox2+, DCX+ and NeuN+ was obtained by dividing the number of EdU+ cells expressing the marker of interest by the total number of EdU+ cells analyzed. The number of EdU+ cells analyzed in DG, SVZ and OB per groups was reported on Table 6.

**Table 6:** Number of EdU+ cells analyzed in DG, SVZ and OB for the study of GFAP+, Sox2+, DCX+ and NeuN+ cells quantification

Marker			Structure			
			DG	SVZ	OB	
GFAP/Sox2	EdU 6 h	CTL	100.6 ± 1.9	210.6 ± 3.5		Supplementary Fig. 2D, E
		SMG	102 ± 0.9	214.7 ± 3.3		
	EdU 24 h	CTL	104.4 ± 0.9	210.2 ± 1.8		
		SMG	104.5 ± 1.8	210.0 ± 4.7		
DCX/NeuN	EdU 7d	CTL	149.4 ± 15.5	104.6 ± 1.2	210.8 ± 1.5	Figs. 4C, E, G and 6C, E, G
		SMG	111.1 ± 14.5	103.6 ± 0.8	211.4 ± 1.2	
		CTL Exe	198.3 ± 17.1	103.1 ± 0.8	208.8 ± 2.9	
		SMG Exe	161.5 ± 16.6	102.7 ± 0.8	209.8 ± 3.9	
	EdU 21d	CTL	72.2 ± 19.2	101.3 ± 1.0	211.2 ± 2.9	Fig. 4C, E, G
		SMG	81.0 ± 1.9	93.5 ± 3.7	209.7 ± 1.1	

### Statistical analysis

All data were averaged across animals within each experimental condition and are presented as mean ± SEM. The normality of the data was checked using Shapiro-Wilk or Kolmogorov–Smirnov test. All statistical analysis were performed with GraphPad Prism 9 (version 9.3.1). Statistical significance was set at *p* < 0.05.

Muscle weight was analyzed using unpaired t-test. The effect of physical exercise on muscle weight was analyzed using two-way ANOVA followed by Tukey test for multiple comparisons. Corticosterone concentration was analyzed using two-way ANOVA to compare CTL and SMG or CTL Exe and SMG Exe groups, and using one-way ANOVA to analyze the time effect in each group. The weight of the rats was analyzed as a ratio (i.e., their weight at D_n_ compared with their weight at the start of the experiment, D0). The evolution of the body weight over time was analyzed using one-sample t-test against the value 1. The difference between CTL and SMG groups was analyzed using two-way ANOVA followed by post-hoc tests for multiple comparisons. The effect of physical exercise was analyzed using three-way ANOVA. Food and water consumption, temperature and glycemia were analyzed using two-way ANOVA. The evolution of the consumption over time was analyzed using Dunnett test for multiple comparisons in relation to D0. The difference between CTL and SMG groups was analyzed using Bonferroni test for multiple comparisons. The effect of physical exercise was analyzed using three-way ANOVA followed by Tukey test for multiple comparisons.

Adult neurogenesis was analyzed using unpaired t-test (or Mann–Whitney test for non-normal data) or using two-way ANOVA followed by Bonferroni test for multiple comparisons. Percentage of EdU/BrdU+ cells expressing GFAP+, Sox2+, DCX+ and NeuN+ was analyzed using paired or unpaired t-test or two- or three-way ANOVA with repeated measures followed by Bonferroni test for multiple comparisons.

### Gene expression study

#### Tissue collection

At the end of the SMG exposure (7d, CTL *n* = 4, SMG *n* = 4, CTL Exo *n* = 4, SMG Exo *n* = 4; 21d, CTL *n* = 4, SMG *n* = 4), rats were deeply anesthetized with an intraperitoneal injection of mixed Exagon (40 mg/kg) and Lurocaïne (20 mg/kg) solution and then perfused transcardially with a 0.9% NaCl solution during 10 min at 13 ml/min. Brains were collected and hippocampi were dissected and placed in tubes containing tri-reagent (TR118, Molecular Center, Inc.). Samples were preserved at −20 °C until use.

#### Preparation of RNA samples for transcriptomic assays

Hippocampus samples were dissected homogenized with BeadBug6 (3 cycles, speed 4350, time 20 s, Benchmak distributed by Dominique Dutscher). Isolation of the total RNA from each hippocampus was performed with Direct-zol RNA miniprep (ZR2053, Zymo) following the supplier procedures. The concentration of RNA was measured by spectrophotometry (NanoDrop 2000, ThermoFisher) for each animal. RNA quality was checked using Lab Chip GX touch HT (PerKin Elmer) and RNA chip (Xmark – High sensitivity). We harmonized the concentration of all sample (~300 ng/µl) and mix the same volume of samples per condition to obtain one RNA solution for each experimental condition.

#### Quantitative real-time Polymerase Chain reaction after transcription (RT-QPCR)

To determine the expression of genes involving in adult neurogenesis, we used the Neurogenesis (SAB Target List) R96 Predesigned 96-well panel (10046994, Bio-Rad). We performed the reverse transcription (RT) reaction with 200 ng/µl of total RNA with the iScript gDNA clear advanced kit (Bio-Rad 172-5035) and the real time qPCR was performed with the Sso Advanced SYBR Green® Supermix (Bio-Rad 172-5270) in the CFX96^TM^ Real-Time System thermocycler (Bio-Rad). All samples were analyzed in triplicates and plate preparations were performed randomly with the same lot of PCR mix. All PCR were made with 40 cycles and the calculated Ct (for quantification) were between 20 and 35 depending on genes. Analyses were performed using CFX Maestro (Bio-Rad). Gene expression was normalized by reference gene (Hprt1), the most stable gene in the context of our study. A change in gene expression was considered only for a modification higher than 2.5-fold compared with the control group.

#### Bulk RNA-sequencing

cDNA libraries were synthesized using 500 ng of RNA from each sample with the « Illumina Stranded mRNA Prep Ligation » kit according to the supplier instructions. Briefly, the first step consisted on capturing mRNAs with magnetic beads targeting their poly-A tail. Then, mRNAs were fragmented, reverse transcribed into double strand cDNA and 3’ adenylated adapters and anchors were ligated to both ends. Finally, the cDNA library was amplified by PCR for 12 cycles and libraries quality was checked with Xmark chip on the Lab Chip GX touch HT (Perkin Elmer). After quantification of individual libraries by q-PCR using the NEBNext library quant kit (New England BioLabs), all the samples were pooled in an equimolar manner. The quality of the pool was checked by capillary gel electrophoresis (Lab Chip GX touch HT, Perkin Elmer) and it showed an average library size of xxxbp and the absence of adapter contamination. The quantity of pool libraries was determined using qPCR with the NEBNext Library Quant Kit for Illumina (contains primers which target the P5 and P7 Illumina adaptor sequences) before sequencing. At 750pM concentration mRNA sequencing was performed using Nextseq 2000 Illumina (paired-end 2 ×100 bp) with a minimum of 30 million reads per sample in the PGTB facility (INRAE - Pierroton).

#### Bioinformatics

Bulk-RNA sequencing reads were first pre-processed to ensure data quality. Thus, fastq files quality was analyzed using the fastp software with default parameters^121^ and only reads longer than 20 bp were kept. Then, reads were aligned to the rat genome (Rnor_6.0 genome assembly) using the splice-aware mapper STAR with default parameters^122^ and gene counts were summarized using the GenCounts options of STAR. To determine differentially expressed genes, we used the DESeq2 algorithm in R with default parameters^123^. Gene expression data were displayed as volcano plots using the EnhancedVolcano package in R (https://github.com/kevinblighe/EnhancedVolcano). Numbers of reads of each sample were indicated in Supplementary Table 1.

### Supplementary information

Supplementary Information

## Data Availability

The datasets generated and analyzed during the current study available from the corresponding author on reasonable request. The transcriptomic datasets generated and analyzed during the current study are available in the NCBI repository (BioProject ID PRJNA1034063), http://www.ncbi.nlm.nih.gov/bioproject/1034063.
