# Supplementary material for: Physical exercise restores adult neurogenesis deficits induced by simulated microgravity
Source: NPJ Microgravity. 2024 Jun 21;10:69. doi: 10.1038/s41526-024-00411-6 (PMC11192769; doi:10.1038/s41526-024-00411-6)
Supplement: Supplementary file 1 — Supplementary Information [file 41526_2024_411_MOESM1_ESM.pdf]

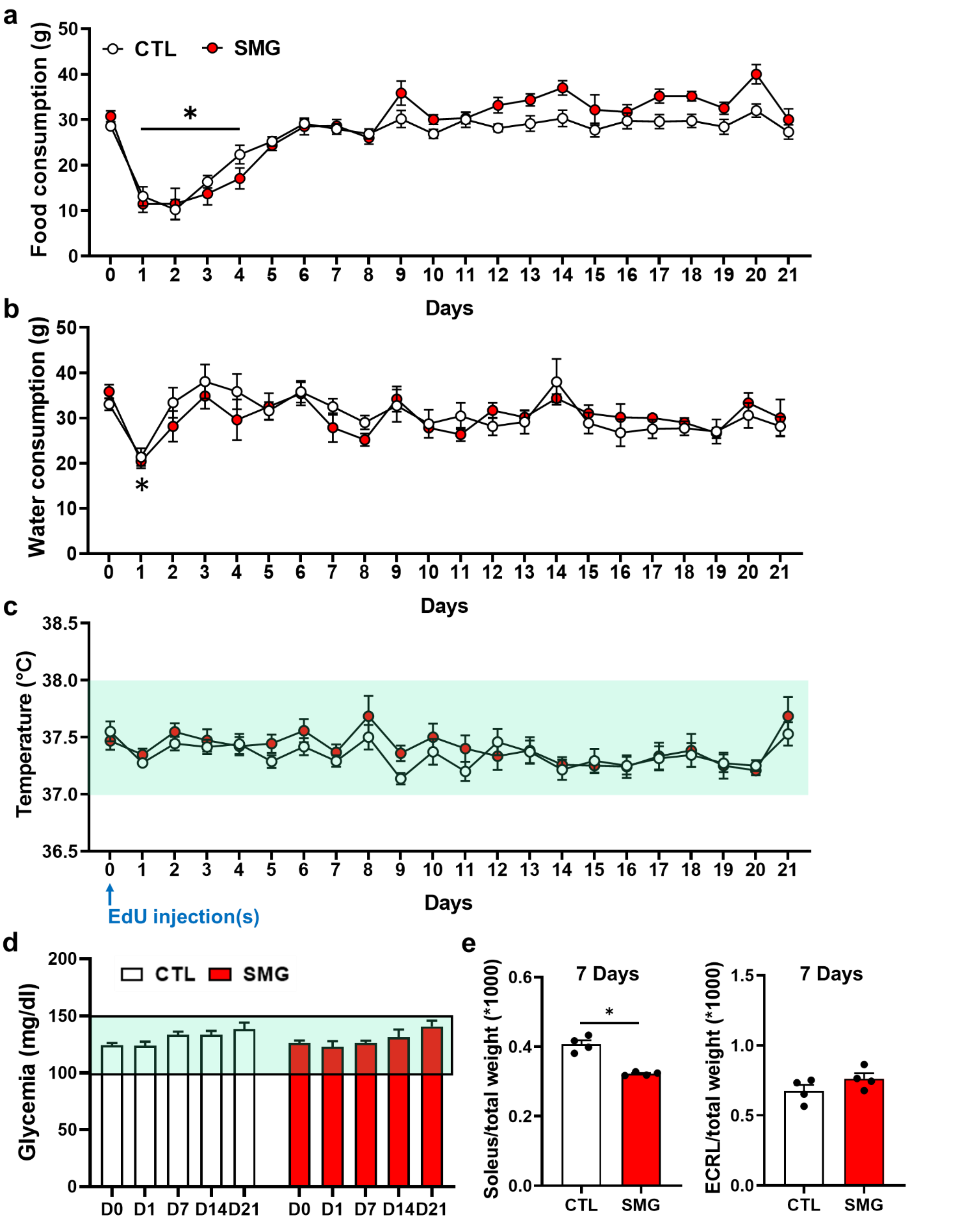

Supplementary Figure 1. Physiological parameters of rats during the adult neurogenesis study. a. Food consumption of the CTL (white) and SMG (red) rats. **b.** Water consumption of the CTL (white) and SMG (red) rats. **c.** Temperature of the CTL (white) and SMG (red) rats. **d.** Glycemia of the CTL (white) and SMG (red) rats at D0, D1, D7, D14 and D21. Green area represents the physiological values. **e.** Weight of the soleus and ECRL muscles related to the total weight of the animals in CTL (white, n = 4) and SMG (red, n = 4) rats. All data are presented as mean ± SEM. * $p < 0.05$.

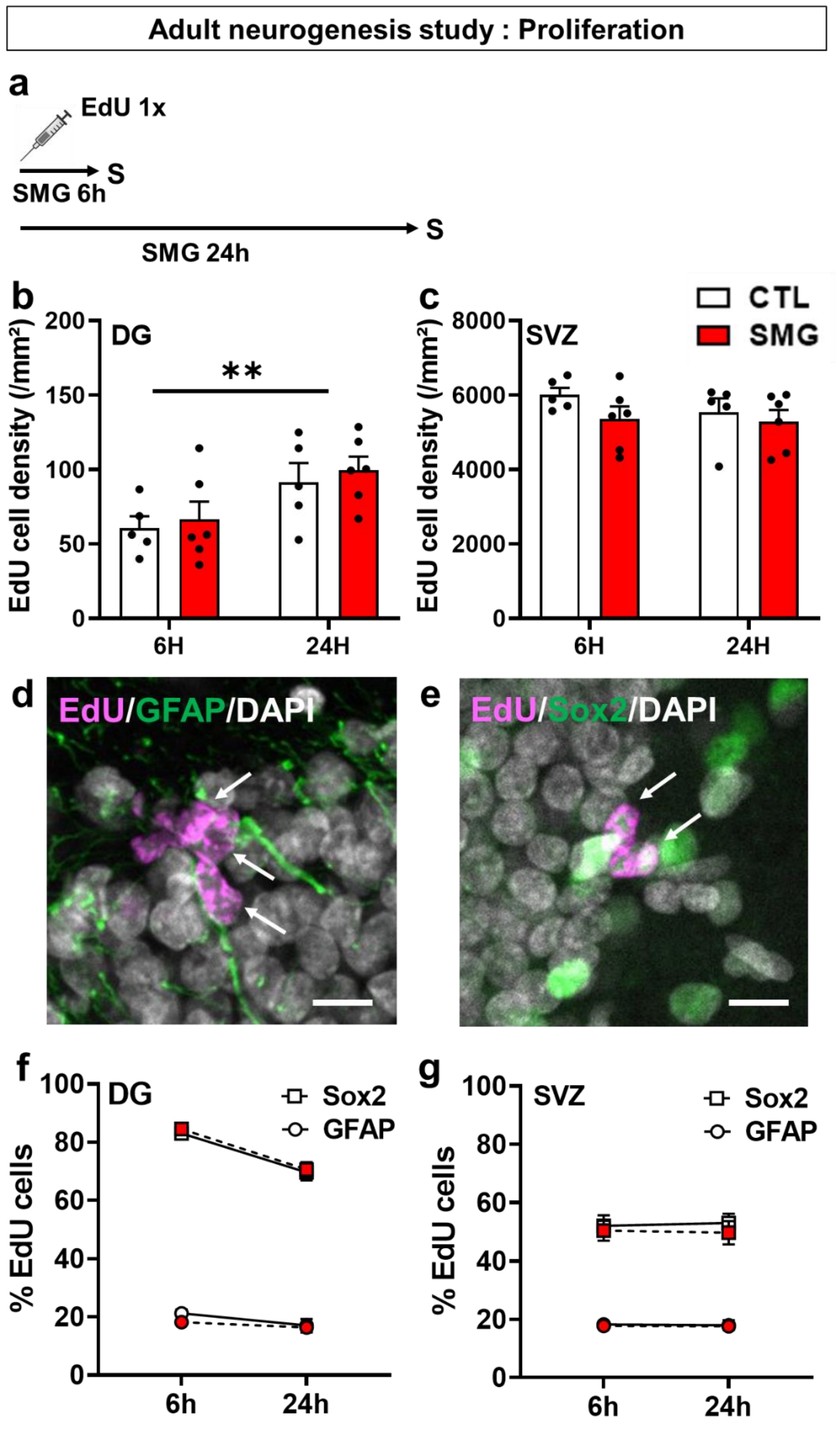

Supplementary Figure 2. Newborn cells proliferation under SMG. a. Experimental design. Rats were injected with EdU and exposed to SMG during 6 or 24 hours. **b.** Density of EdU cells per mm² in the DG of CTL (white, 6H: n = 5; 24H: n = 5) and SMG (red, 6H: n = 6; 24H: n = 6) rats. **c.** Density of EdU cells per mm² in the SVZ of CTL (white, 6H: n = 5; 24H: n = 5) and SMG (red, 6H: n = 6; 24H: n = 6) rats. **d.** Example of EdU+ cells expressing GFAP in the DG of the hippocampus. Scale bar: 10µm. **e.** Example of EdU+ cells expressing Sox2 in the DG of the hippocampus. Scale bar: 10 µm. **f.** Percentage of EdU cells expressing GFAP or Sox2 in the DG of CTL (white, 6H: n = 5; 24H: n = 5) and SMG (red; 6H: n = 6; 24H: n = 6) rats. **g.** Percentage of EdU cells expressing GFAP or Sox2 in the SVZ of CTL (white, 6H: n = 5; 24H: n = 5) and SMG (red, 6H: n = 6; 24H: n = 6) rats. All data are presented as mean ± SEM. ** $p < 0.01$.

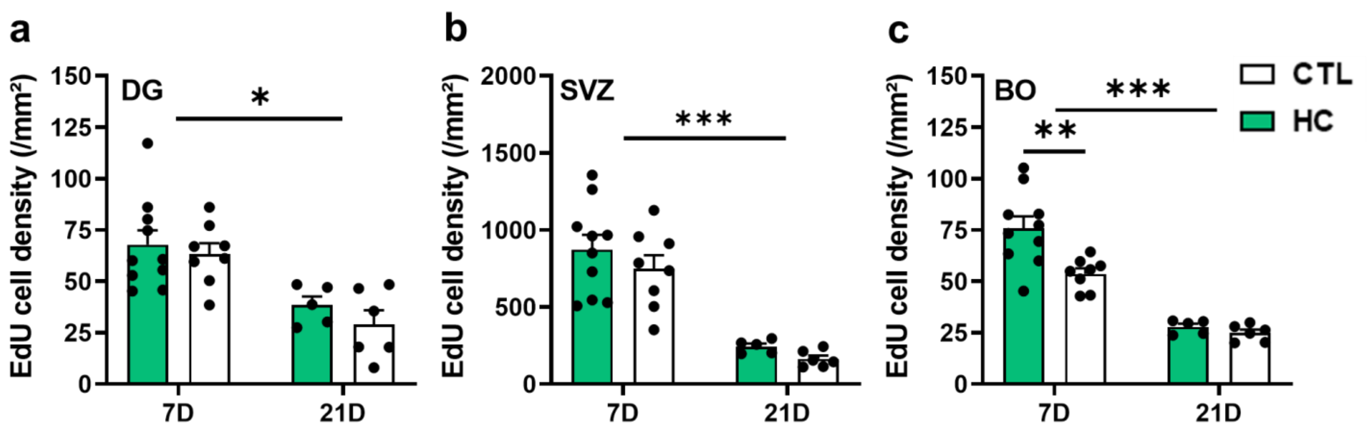

Supplementary Figure 3. Newborn cells survival in single-housed (CTL) and grouped-housed rats (HC). a. Density of EdU cells per mm² in the DG of HC (green, 7D: n = 10; 21D: n = 5) and CTL (white, 7D: n = 8; 21D: n = 6) rats. No difference was observed between HC and CTL rats (two-way ANOVA, SMG effect $F(1, 25) = 1.03$, $p = 0.32$). **b.** Density of EdU cells per mm² in the SVZ of HC (green, 7D: n = 10; 21D: n = 5) and CTL (white, 7D: n = 8; 21D: n = 6) rats. No difference was observed between HC and CTL rats (two-way ANOVA, SMG effect $F(1, 25) = 1.41$, $p = 0.25$). **c.** Density of EdU cells per mm² in the OB of HC (green, 7D: n = 10; 21D: n = 5) and CTL (white, 7D: n = 8; 21D: n = 6) rats. A significant effect was observed between HC and CTL rats at D7 (two-way ANOVA, SMG effect $F(1, 25) = 7.76$, $p = 0.01$; Bonferroni multiple comparisons test: 7D $p = 0.003$, 21D $p > 0.99$). All data are presented as mean ± SEM. * $p < 0.05$, ** $p < 0.01$, *** $p < 0.005$.

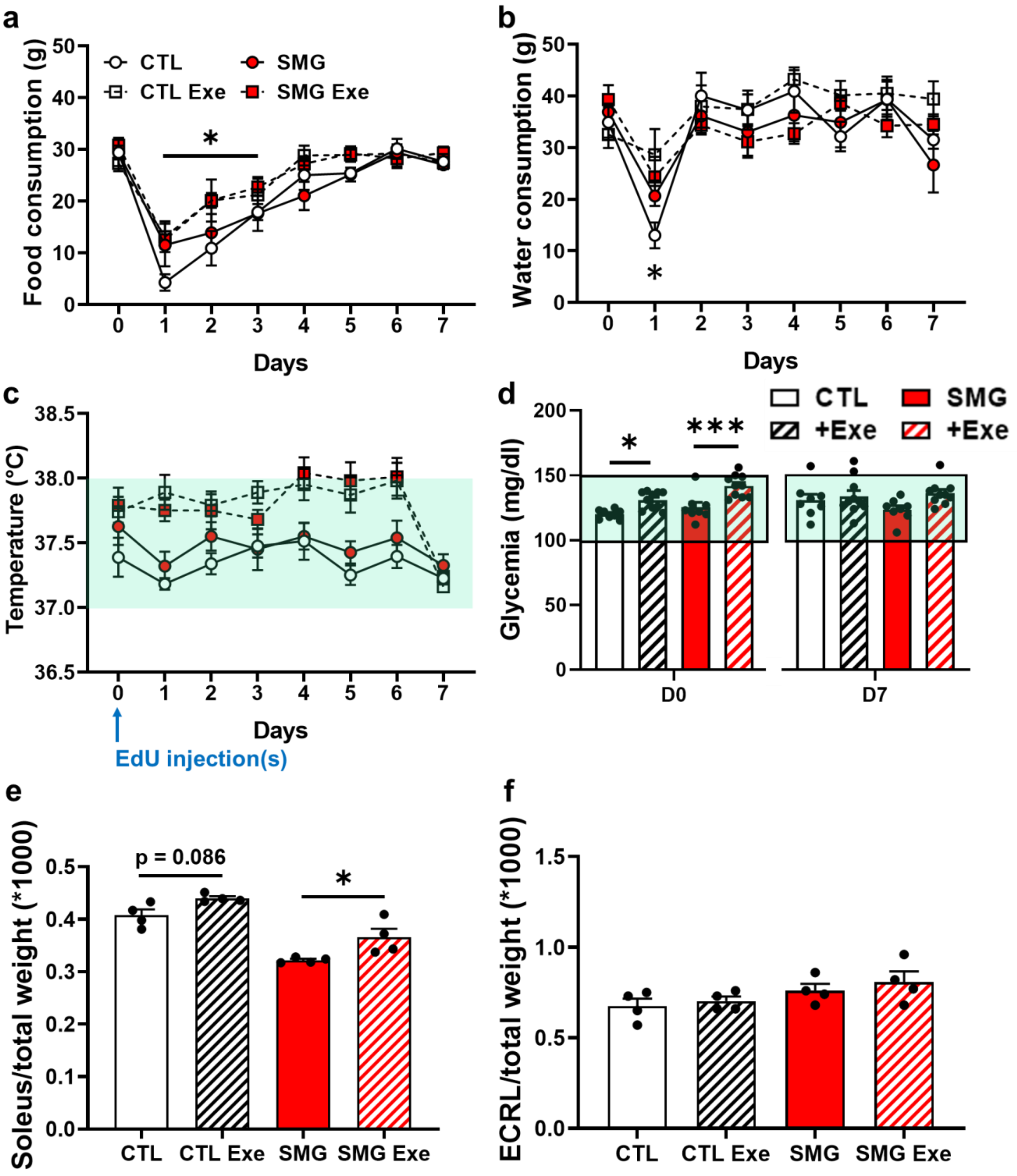

Supplementary Figure 4. Physiological parameters of rats during the adult neurogenesis study with physical exercise. a. Food consumption of the CTL (white, CTL: n = 8; CTL Exe: n = 10) and SMG (red, SMG: n = 8; SMG Exe: n = 10) rats exposed (scare) or not (circle) to physical exercise. **b.** Water consumption of the CTL (white, CTL: n = 8; CTL Exe: n = 10) and SMG (red, SMG: n = 8; SMG Exe: n = 10) rats exposed (scare) or not (circle) to physical exercise. **c.** Temperature of the CTL (white, CTL: n = 8; CTL Exe: n = 10) and SMG (red, SMG: n = 8; SMG Exe: n = 10) rats exposed (scare) or not (circle) to physical exercise. **d.** Glycemia of the CTL (white, CTL: n = 8; CTL Exe: n = 10) and SMG (red, SMG: n = 8; SMG Exe: n = 10) rats exposed or not to physical exercise at D0 and D7. **e.** Weight of the soleus muscle related to the total weight of the animals in CTL (white, n = 4) and SMG (red, n = 4) rats. **f.** Weight of the ECRL muscle related to the total weight of the animals in CTL (white, n = 4) and SMG (red, n = 4) rats. All data are presented as mean ± SEM. * $p < 0.05$.

Supplementary table S1: numbers of reads for all samples included in the statistical analysis

obs	sample	#raw _reads	#trimmed_reads	#mapped_reads
value	JL145_S22	15110985	15071917	12646752
value	JL146_S23	32243364	32168835	27239379
value	JL147_S24	22802544	22746694	18714593
value	JL148_S25	26605297	26534670	22580291
value	JL149_S26	25021439	24966339	20969856
value	JL150_S27	13996770	13964595	11271234
value	JL151_S28	26375688	26304944	21173415
value	JL152_S36	17494248	17453632	14297653
value	JL153_S30	24762396	24700168	20165503
value	JL154_S31	23187645	23122244	17902700
value	JL155_S32	26972093	26911460	22587504
value	JL156_S33	29370012	29301028	24512183
value	JL157_S34	24570493	24514049	20564860
value	JL158_S35	18983014	18925278	14549338
value	JL159_S29	20573140	20518984	16291833
value	JL160_S37	27903407	27833016	22479178
value	JLR57_S14	17893088	17849761	15055516
value	JLR58_S15	24857958	24794206	20826156
value	JLR59_S16	31416968	31346116	26460494
value	JLR60_S17	29524702	29463392	25335566
value	JLR61_S18	24043444	23989070	20117735
value	JLR68_S19	19051593	19009665	16282521
value	JLR69_S20	19037911	18997241	16428164
value	JLR70_S21	20378520	20332908	17533073
